# A transthyretin monomer intermediate undergoes local unfolding and transient interaction with oligomers in a kinetically concerted aggregation pathway

**DOI:** 10.1016/j.jbc.2022.102162

**Published:** 2022-06-18

**Authors:** Xun Sun, James A. Ferguson, H. Jane Dyson, Peter E. Wright

**Affiliations:** Department of Integrative Structural and Computational Biology and Skaggs Institute of Chemical Biology, The Scripps Research Institute, La Jolla, California, USA

**Keywords:** transthyretin aggregation, amyloidogenic proteins, aggregation kinetics, real-time NMR, exchange broadening, local unfolding, monomeric intermediate, oligomeric intermediate, BTFA, 3-bromo-1,1,1-trifluoroacetone, DOSY, diffusion-ordered NMR spectroscopy, HSQC, ^1^H,^15^N-heteronuclear single quantum coherence, MES, 4-morpholineethansulfonic acid, TTR, transthyretin

## Abstract

Transthyretin (TTR) amyloidosis is associated with tissue deposition of TTR aggregates. TTR aggregation is initiated by dissociation of the native tetramer to form a monomeric intermediate, which locally unfolds and assembles into soluble oligomers and higher-order aggregates. However, a detailed mechanistic understanding requires kinetic and structural characterization of the low population intermediates formed. Here, we show that the monomeric intermediate exchanges with an ensemble of oligomers on the millisecond timescale. This transient and reversible exchange causes broadening of the ^19^F resonance of a trifluoromethyl probe coupled to the monomeric intermediate at S85C. We show the ^19^F linewidth and *R*_2_ relaxation rate increase with increasing concentration of the oligomer. Furthermore, introduction of ^19^F probes at additional TTR sites yielded distinct ^19^F chemical shifts for the TTR tetramer and monomer when the trifluoromethyl probe was attached at S100C, located near the same subunit interface as S85C, but not with probes attached at S46C or E63C, which are distant from any interfaces. The ^19^F probe at E63C shows that part of the DE loop, which is solvent accessible in the tetramer, becomes more buried in the NMR-visible oligomers. Finally, using backbone amides as probes, we show that parts of the EF helix and H-strand become highly flexible in the otherwise structured monomeric intermediate at acidic pH. We further find that TTR aggregation can be reversed by increasing pH. Taken together, this work provides insights into location-dependent conformational changes in the reversible early steps of a kinetically concerted TTR aggregation pathway.

The human transthyretin (TTR) tetramer transports thyroxine and holoretinol-binding protein in plasma and cerebrospinal fluid (1) and is the causative agent for TTR amyloidosis (2). Each TTR protomer consists of two four-stranded β-sheets (strands D-A-G-H and C-B-E-F) and a short (EF) helix. Aberrant aggregation of WT TTR causes TTR amyloidosis (ATTRwt), which manifests as a late-onset cardiomyopathy that affects as many as one in four people over age 80 (3). Mutations that destabilize tetrameric TTR result in familial TTR amyloidosis (ATTR) and lead to earlier onset of amyloid polyneuropathy and cardiomyopathy (4, 5). The initial step of TTR aggregation involves dissociation of the TTR tetramer (T) to form a monomeric intermediate (M), which subsequently self-assembles into oligomers (O) and eventually higher-order aggregates (A) (1, 6). A quantitative understanding of how the dissociation and aggregation equilibria are coupled is of great importance to provide insight into the molecular determinants in early steps of the TTR aggregation pathway.

We previously developed an efficient 1D ^19^F-NMR aggregation assay to monitor TTR aggregation by labeling TTR with a 3-bromo-1,1,1-trifluoroacetone (BTFA) probe at a strategic site (S85C, labeled protein denoted as S85-TTR^F^) that reports on distinct ^19^F chemical shifts for the T, M, and NMR-visible O species (7). Based on a simple linear kinetic model, T⇋M ⇋A or T⇋M⇋O⇋A (if NMR-visible O aggregation intermediates are observable), we have quantified the apparent equilibrium constants and the associated free energy change (7). However, the structural details of the two aggregation-prone intermediates, M and NMR-visible O, remained elusive. In this work, we introduce three additional sites for the ^19^F-BTFA labeling (Figs. 1 and S1). The use of the highly sensitive ^19^F probe enables direct observation of NMR-visible O species with estimated molecular weights larger than ∼400 kDa. The ^19^F spectra reveal distinct location-dependent solvent accessibilities of the trifluoromethyl probe. The TTR aggregation rate constants are similar regardless of the location of the ^19^F probe, and the trace of ^19^F-NMR signal decay closely mirrors the increase in optical turbidity for each ^19^F-bearing mutant. Moreover, we find that M transiently and reversibly exchanges with soluble oligomers, resulting in broadening of the ^19^F signal of M and NMR-visible O in S85-TTR^F^. We have further expanded the range of aggregation probes to backbone amides using standard 2D-NMR experiments with ^15^N-labeled TTR. These studies show that kinetically concerted early steps in TTR aggregation are reversible and reveal enhanced conformational flexibilities in parts of the EF helix and the H strand in the monomeric intermediate.

## Results

### ^19^F chemical shifts of the BTFA-labeled TTR variants

**Figure 1:**
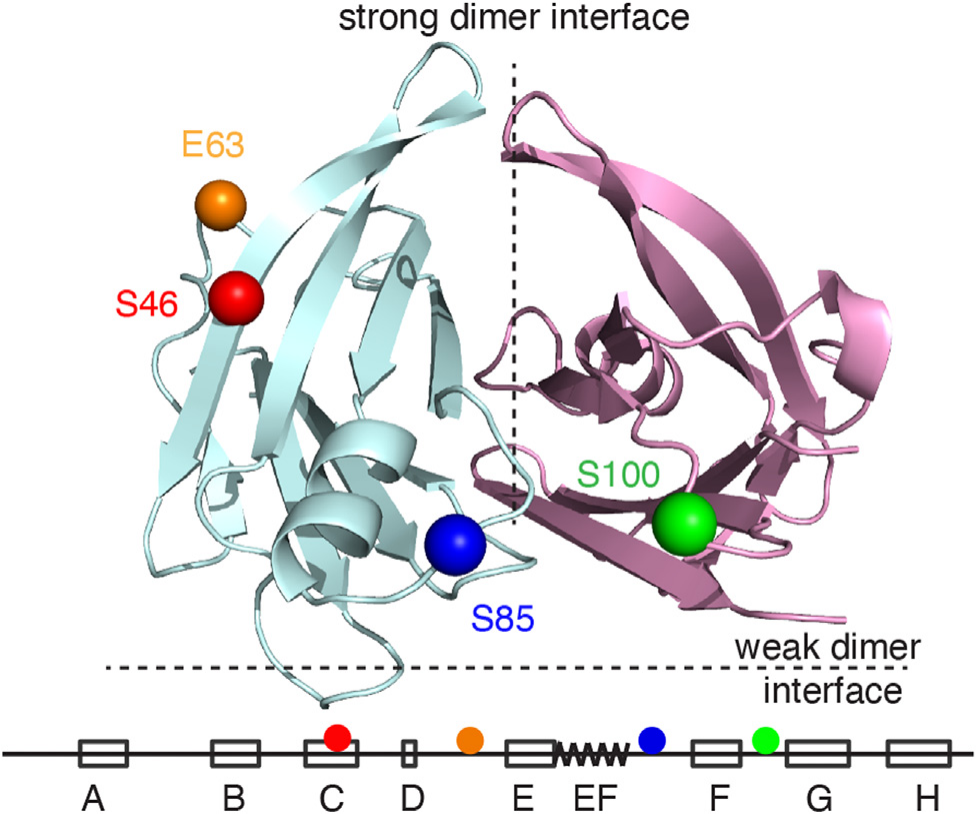
**Locations of the four**^**19**^**F-BTFA probes.** Residues at the four introduced Cys sites are shown on the structure of a TTR dimer (PDB: 5CN3). Three sites (S46, E63 and S85) are shown as Cα spheres on the left protomer (*light blue*) and the Cα sphere of S100 is showed on the right protomer (*pink*). The locations of the strong and weak dimer interfaces in the tetramer are indicated. The locations of the introduced Cys sites in the tetramer are shown in Fig. S1. The secondary structure of TTR, including the introduced Cys sites, is shown schematically below the TTR structure. BTFA, 3-bromo-1,1,1-trifluoroacetone; PDB, Protein Data Bank; TTR, transthyretin.

Our previous real-time ^19^F NMR study of TTR aggregation at pH 4.4 (7) used the mutant C10S-S85C coupled to BTFA (S85-TTR^F^), where the CF_3_ probe was located in the EF loop (residues 81–91) adjacent to the strong dimer interface (Figs. 1 and S1). To report on the aggregation process from other sites, we introduced the BTFA probe at S46C (C strand, residues 39–51, denoted S46-TTR^F^), E63C (DE loop, residues 56–67, E63-TTR^F^) and S100C (FG loop, residues 97–103, S100-TTR^F^), all within a C10S background. Like S85C, S100C is also adjacent to the strong dimer interface, whereas S46C and E63C are on the opposite side of the TTR protomer (Figs. 1 and S1).

**Figure 2:**
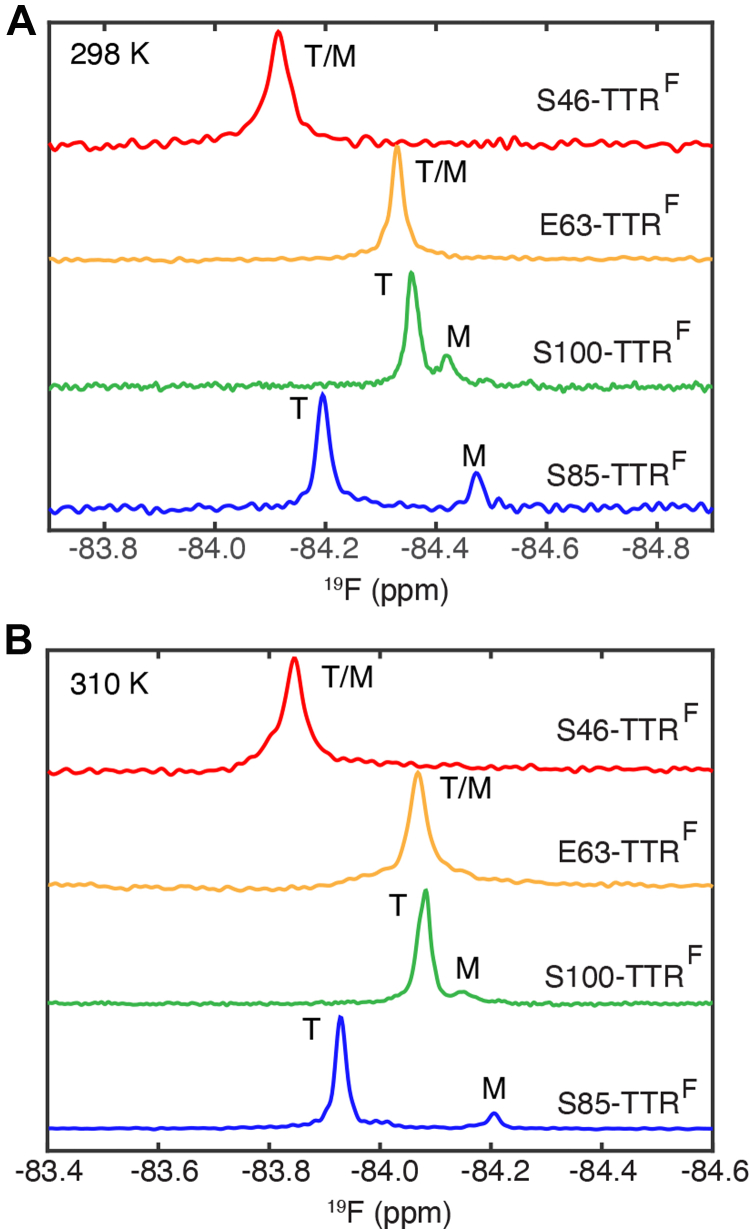
^**19**^**F NMR spectra of BTFA probes located at distinct sites.** Spectra of the four BTFA-labeled TTR constructs (10 μM, pH 4.4) at 298 K (*A*) and 310 K (*B*) after a pseudoequilibrium of all aggregating TTR species was achieved. The maximal peak height was normalized for comparison. ^19^F resonances are labeled T (tetramer) and M (monomer) or as T/M when the two resonances are presumed to be overlapped. Data for S85-TTR^F^ were previously reported in Ref (7). BTFA, 3-bromo-1,1,1-trifluoroacetone; TTR, transthyretin.

Figure 2 shows the 1D ^19^F NMR spectra of the four BTFA-labeled TTR proteins at pH 4.4 and 298 K, where the WT tetramer begins to dissociate into monomers and aggregate (8). Under these conditions, two resolved ^19^F resonances are observed for S85-TTR^F^ and S100-TTR^F^, but only one peak is observed for S46-TTR^F^ or E63-TTR^F^ (Fig. 2*A*). For S100-TTR^F^, the ^19^F-NMR diffusion-ordered NMR spectroscopy (DOSY) experiment shows that the translational diffusion coefficient (*D*) of the upfield minor resonance is 1.6 ± 0.1 times larger than that of the major downfield peak (Fig. S2), consistent with the ratio of *D* values for the M and T states predicted using the Stokes–Einstein equation (1.53) and with our previous ^19^F-NMR measurement for S85-TTR^F^ (1.6 ± 0.2 (7)). Therefore, we assigned the upfield and downfield resonances in the S100-TTR^F^ spectrum to the M and T states, respectively.

There is a larger difference between the ^19^F-NMR chemical shifts of T and M in S85-TTR^F^ (∼0.28 ppm) than in S100-TTR^F^ (∼0.06 ppm). In both cases, the upfield shift of the M resonance indicates that the probe is more solvent exposed than in T. By contrast, the ^19^F chemical shifts of T and M in S46-TTR^F^ and E63-TTR^F^ are likely degenerate since the probes are distant from the subunit interfaces and both are solvent exposed even in T. We therefore designate the single peak observed for these probes as T/M in Figure 2*A*. At pH 7, the ^19^F resonances of S46-TTR^F^ and E63-TTR^F^ have similar chemical shifts (−84.4 and −84.3 ppm respectively, Fig. S3); upon lowering the pH to 4.4, the S46-TTR^F^ peak is shifted strongly downfield, likely reflecting protonation of H31, which packs against S46 in the TTR structure. At pH 4.4 and 298 K, the linewidth of the ^19^F resonance in the spectrum of S46-TTR^F^ (24 Hz) is larger than those of the other three mutants (12–16 Hz). These differences likely arise from probe location: S46 is located in the C-strand, whereas the other probes are on more flexible loops (Fig. 1). The ^19^F spectra of each derivative are similar at 310 K (Fig. 2*B*), but the higher temperature decreases the M population of both S85-TTR^F^ and S100-TTR^F^.

### Aggregation kinetics of BTFA-labeled TTR variants

**Figure 3:**
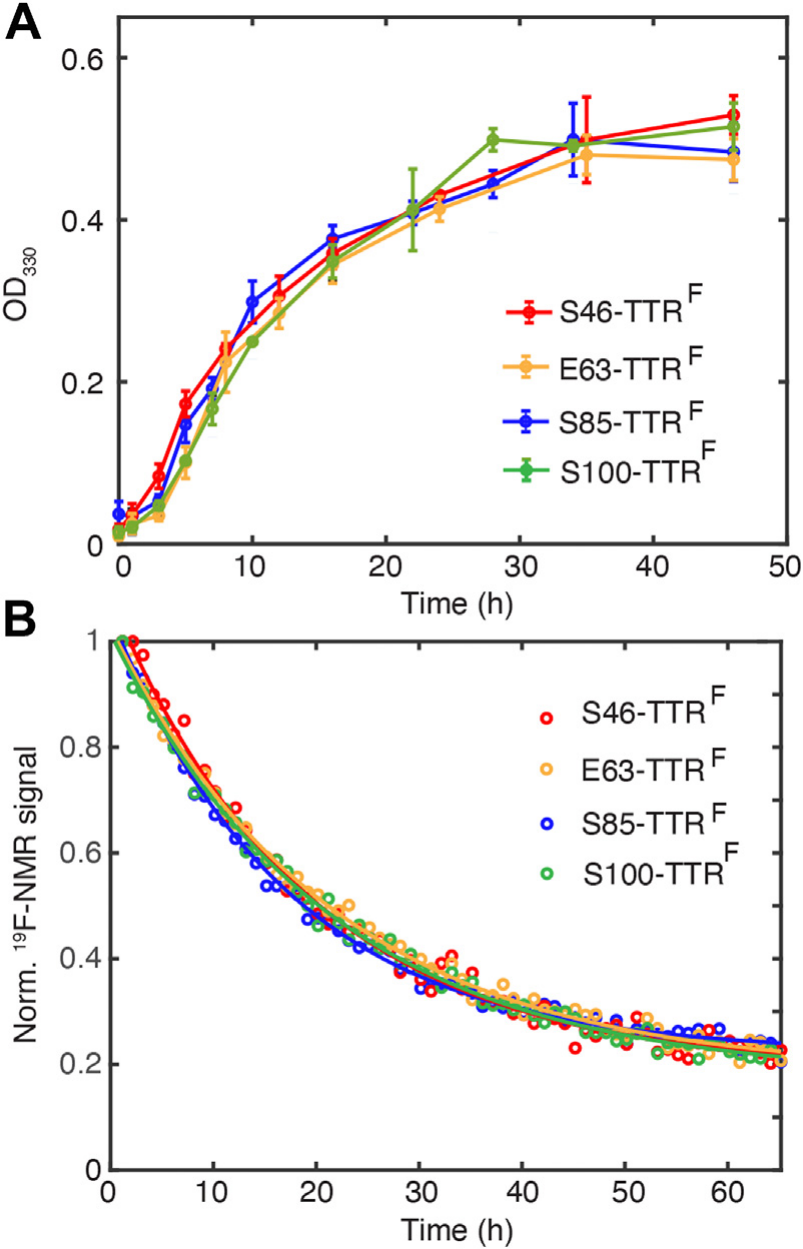
**Aggregation kinetics measured by**^**19**^**F-NMR and turbidity.***A*, change in turbidity at 330 nm (*A*_330_) of the four ^19^F-BTFA labeled TTR constructs (10 μM) following initiation of aggregation at pH 4.4 and 310 K. The error bars represent one SD from three independent measurements. *B*, decay of the total ^19^F-NMR peak area for the four ^19^F TTR constructs (10 μM) at pH 4.4 and 310 K. For S85-TTR^F^ and S100-TTR^F^, the signal from T and M is combined. The first data point was set to 1.0 in each mutant for normalization. The solid curves are the single exponential fits (0.058, 0.052, 0.046, and 0.047 h^−1^ for S85-TTR^F^, S46-TTR^F^, E63-TTR^F^, and S100-TTR^F^, respectively). The data of S85-TTR^F^ were replotted from Ref (7) for comparison. BTFA, 3-bromo-1,1,1-trifluoroacetone; TTR, transthyretin.

We compared the aggregation kinetics of all four BTFA-bearing mutants at physiological temperature (310 K) and concentration (10 μM). The time-dependent turbidity traces (*A*_330_) at pH 4.4 and 310 K are nearly superimposable (Fig. 3*A*), suggesting that the kinetics of forming aggregates that are sufficiently large to scatter light are independent of the locations of the BTFA probes. Formation of these large particles leads to time-dependent loss of the ^19^F NMR signal (Fig. 3*B*), which undergoes single-exponential decay with similar rate constants (∼0.05–0.06 h^−1^) for all mutants. The formation of ^19^F-NMR-invisible species closely follows the increase in *A*_330_ (Fig. S4). The observation of similar aggregation rates regardless of the location of the ^19^F probe confirms that the Cys mutations, and BTFA labeling do not alter the aggregation process.

The ability to resolve both the T and M resonances of S100-TTR^F^ allowed deconvolution of the individual aggregation steps based on the three-state kinetic model (T⇋M⇋A) derived for S85-TTR^F^ (7). The fitted rate constants are similar for both the S85C and S100C constructs (Table S1 and Fig. S5). The slow relaxation rate constants (0.06 h^−1^) closely mirror the apparent rate constants from the single exponential fits of the combined signal loss of the T and M resonances (Fig. 3*B*).

### ^19^F chemical shifts of NMR-visible oligomers

**Figure 4:**
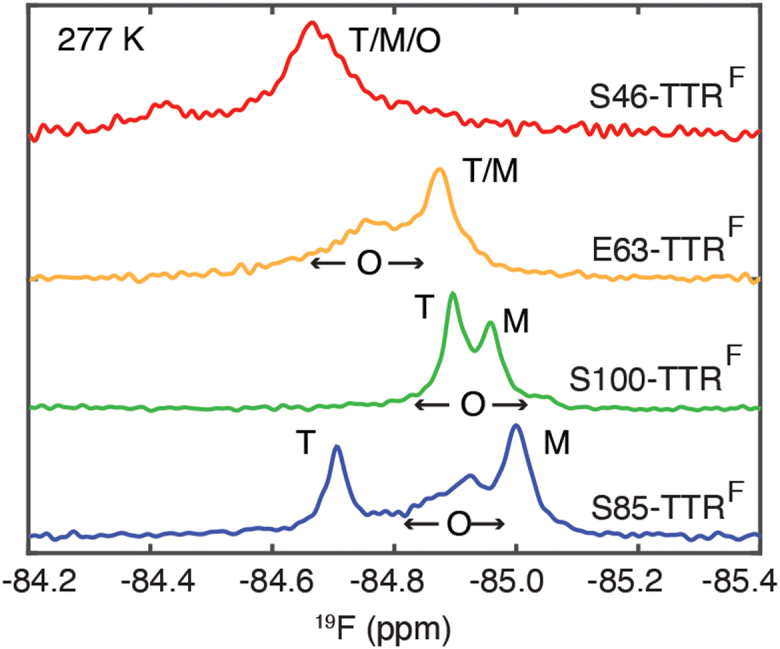
**Oligomer formation at low temperature.**^19^F-NMR spectra of the four BTFA-labeled TTR constructs measured at 10 μM at pH 4.4 at 277 K after a pseudoequilibrium of all aggregating TTR species was achieved. The maximal peak height was normalized for comparison. ^19^F resonances are labeled with T (tetramer), M (monomer), or O (NMR-visible oligomer) where applicable. BTFA, 3-bromo-1,1,1-trifluoroacetone; TTR, transthyretin.

During aggregation of S85-TTR^F^ at pH 4.4 and 277 K, monomeric (M) and NMR-visible oligomeric (O) intermediates accumulate, with O giving rise to a broad ^19^F peak (∼84 Hz linewidth) between the T and M resonances (22–24 Hz linewidth, Fig. 4). The location of the O peak between T and M indicates that the S85C-BTFA probe in the oligomers is less solvent exposed than in M but more so than in T. For S46-TTR^F^, only a single broad ^19^F resonance (∼81 Hz linewidth) is observed; constituent T, M, or O peaks could not be resolved and are likely overlapped. For S100-TTR^F^, it is likely that the ^19^F resonance of O lies under the broad and overlapped peaks arising from T and M since the time-dependent trace of overall NMR signal loss is comparable to that of S85-TTR^F^ (Fig. S6). Interestingly, for E63-TTR^F^, a very broad shoulder (∼110 Hz linewidth) is observed downfield of the ^19^F peak arising from T + M (∼32 Hz linewidth), indicating that the CF_3_ probe in the DE loop has lower solvent exposure, that is, is more buried, in O than in T (9). This result contrasts with that for the CF_3_ probe at S85C, which exhibits greater exposure to solvent in O than in T.

### Enhanced ^19^F *R*_2_ relaxation rate of M resonances due to exchange with oligomeric species

**Figure 5:**
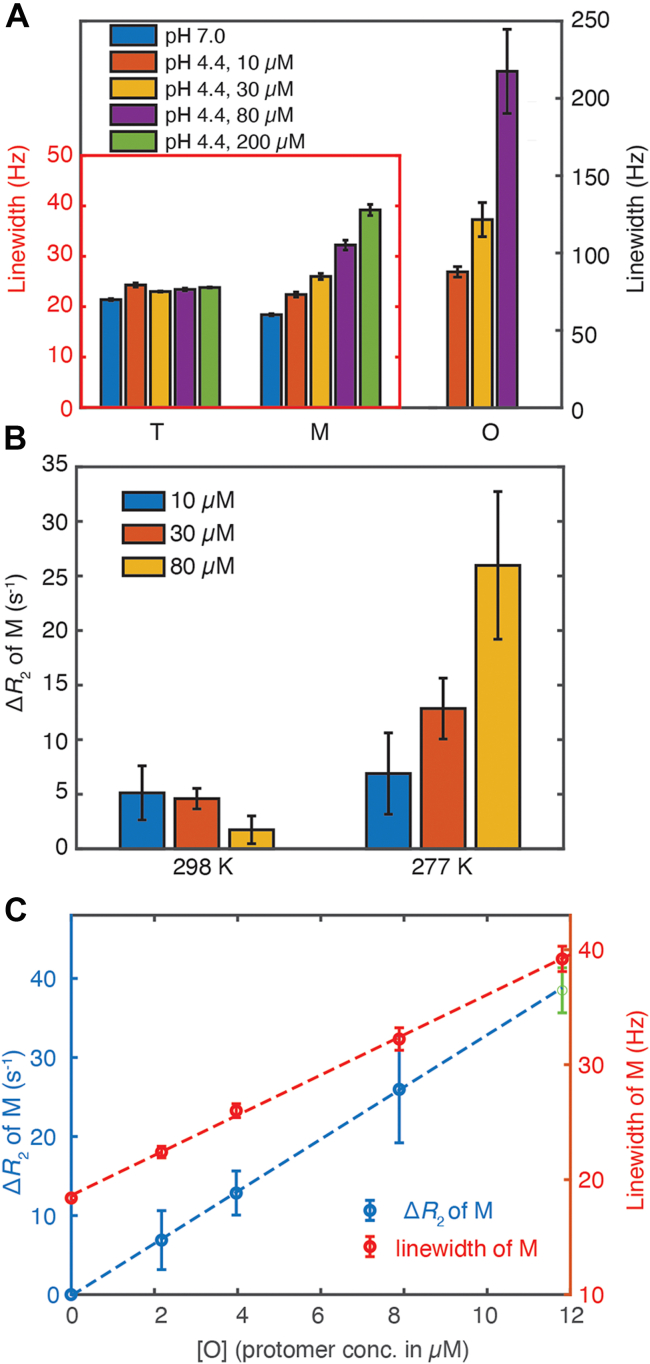
**Linewidth and R**_**2**_**Relaxation of S85-TTR**^**F**^**.***A*, Lorentzian linewidths of the ^19^F resonances of T, M, and O of S85-TTR^F^ at varying TTR concentrations (10, 30, 80, and 200 μM) at pH 4.4 and 277 K in aggregation buffer. Because the M resonance does not appear in the spectrum of S85-TTR^F^ at pH 7.0, the linewidths of the M peak at pH 7.0 were measured in the ^19^F spectrum of S85-TTR^F^-F87A, which populates both M and T states at this pH (10). The smaller linewidths of M and T are shown in a *red box* on the left axis and the larger linewidth of O is shown on the *black* right axis. *B*, concentration-dependent changes at 277 K in ^19^F *R*_2_ relaxation rate constants, relative to the *R*_2_ for the M species of S85-TTR^F^-F87A at pH 7.0 (Δ*R*_2_ = *R*_2 (pH 4.4)_ – *R*_2 (pH 7.0)_), for the M peak of S85-TTR^F^ at pH 4.4 from 10 to 80 μM. Δ*R*_2_ of M is not as sensitive to the total TTR concentrations between 10 and 80 μM at 298 K as 277 K. Error bars in panels (*A*) and (*B*) are fitting uncertainties, estimated as one SD from 50 bootstrapped datasets. *C*, positive linear correlations between the protomer concentration of NMR-visible O species and Δ*R*_2_ (*blue*, on the left axis) as well as the linewidth of M (*red*, on the right axis). The linewidth of the M peak at pH 7.0 from the ^19^F spectrum of S85-TTR^F^-F87A is plotted at [O] = 0 μM, where Δ*R*_2_ = 0 s^−1^. The Δ*R*_2_ value with 200 μM S85-TTR^F^ (*green*) is extrapolated based on the linear correlation between the *R*_2_ and linewidth of M (Fig. S7*H*). The *dashed blue line* is a linear fit between Δ*R*_2_ and [O] without the 200 μM *R*_2_, and the *dashed red line* is a linear fit between linewidth and [O]. In both cases the correlation coefficients are greater than 0.999. The slope of Δ*R*_2_ against [O] is 3.3 × 10^6^ M^−1^ s^−1^ with or without the 200 μM Δ*R*_2_ used in the fitting. TTR, transthyretin.

Despite the molecular weight difference between M and T, their ^19^F resonances have similar linewidths in spectra of S85-TTR^F^ and S100-TTR^F^ at pH 4.4 (Table S2), suggesting possible line broadening of M under conditions where aggregation occurs. We focused on S85-TTR^F^ where the T and M resonances are better resolved. The linewidth of the M resonance increases upon lowering the pH at 277 K from 7.0 (nonaggregating; linewidth of M measured for S85-TTR^F^-F87A, which has a mixed population of M and T at pH 7.0 (10), conditions under which the population of the M species of S85-TTR^F^ is small and no M peak can be observed) to pH 4.4 (aggregating) (Fig. 5*A*). Increasing the total concentration of S85C-BTFA at pH 4.4 also increases the linewidth of the M peak. In contrast, the linewidth of the T peak at 277 K is largely independent of pH and TTR concentration (Fig. 5*A*). We also observed a consistent, concentration-dependent increase in the ^19^F *R*_2_ relaxation rate of M at 277 K/pH 4.4 relative to that at 277 K/pH 7.0 (Δ*R*_2_ = *R*_2 (pH 4.4)_ – *R*_2 (pH 7.0)_ > 0, where the reference *R*_2_ is that of the M species of S85-TTR^F^-F87A at pH 7.0) (Fig. 5*B*). By contrast, the *R*_2_ relaxation rate of the T resonance at 298 K and 277 K is independent of both concentration and pH (Fig. S7*A*).

Since neither the ^19^F linewidth nor the *R*_2_ relaxation rate constant of T increases systematically with concentration, it is unlikely that increased solution viscosity at higher total TTR concentrations (up to 200 μM) could be responsible for the elevated ^19^F Δ*R*_2_ for M. A positive Δ*R*_2_ of M was also observed at 298 K/pH 4.4 (Fig. 5*B*), conditions under which ^19^F resonances associated with the NMR-visible O species are not observed (Fig. 2*A*). As shown previously (7), the loss of overall ^19^F signal indicates the presence of high molecular weight, NMR-invisible species. The absence of positive correlation between Δ*R*_2_ and monomer concentration (Fig. S7*B*) shows that the enhanced relaxation does not arise from self-association of M to form dimer or tetramer. Given that interconversion between T and M at 298 K/pH 4.4 (7) is very slow (on a timescale of hours), the increased *R*_2_ of M at 298 K/pH 4.4 is likely due to transient exchange between M and NMR-invisible species. The observed ^19^F *R*_2_ is not dependent on how long the S85-TTR^F^ sample is allowed to aggregate (Fig. S7*C*), yet the turbidity increases during this time (Fig. S7*D*), signifying continuous formation of large insoluble particles. Taken together, these two observations rule out the possibility that the observed Δ*R*_2_ of M is due to exchange with large, insoluble aggregates; if it were, the Δ*R*_2_ of M would be expected to increase over time. We therefore suggest that at 298 K/pH 4.4, free M in solution exchanges with a pseudo–steady-state concentration of soluble, high molecular weight, NMR-invisible oligomers but does not transiently exchange with the insoluble aggregates that contribute to increasing turbidity.

The ^19^F Δ*R*_2_ values of M at 277 K/pH 4.4 are much larger than at 298 K/pH 4.4 and are more strongly dependent on the total S85-TTR^F^ concentration (Fig. 5*B*). The larger Δ*R*_2_ at 277 K/pH 4.4 is not caused by exchange between M and large insoluble aggregates, as turbidity at 277 K/pH 4.4 is much less than that at 298 K/pH 4.4 (Fig. S7, *D*–*E*). The increased Δ*R*_2_ values do not simply result from overlap with broad resonances associated with the NMR-visible O species: the decay of M peak intensities in the *R*_2_ measurements is well fitted by a single exponential, which would not be expected if there was substantial spectral overlap. Both the linewidth and Δ*R*_2_ of the M resonance exhibit a linear correlation with the concentration of NMR-visible oligomer ([O], expressed as protomer concentration) (Fig. 5*C*). This correlation is attributed to an increase in the population of available M-binding sites in the NMR-visible oligomer (${\left[\text{O}\right]}_{\text{free}}$) with increasing TTR concentration, resulting in increased $k_{\text{on}}^{\text{app}}$ (= $k_{\text{on}}{\left[\text{O}\right]}_{\text{free}}$). Exchange between M and NMR-visible O species is sufficient to account for the observed increase in Δ*R*_2_ of M at 277K; this is confirmed by numerical simulations of the Bloch–McConnell equation (11, 12) for two-state exchange between free M (F state) and a state in which M is bound to soluble oligomers (B state, Fig. S7*F*). Millisecond timescale exchange between alternative conformations of M with distinct ^19^F chemical shifts could potentially contribute to the increased linewidth of M. However, we observed no changes in the peak intensity for M upon varying the Carr–Purcell–Meiboom–Gill pulsing frequency from 2000 to 4000 s^−1^ at 277 K/pH 4.4 with 80 μM total TTR concentration (Fig. S7*G*), indicating that conformational exchange within M does not contribute to the *R*_2_ relaxation measurements with a 4000 s^−1^ pulsing rate.

The exchange between M and the NMR-visible oligomers also broadens the ^19^F linewidth of O as a function of the M concentration (Fig. S8*A*). ^19^F-DOSY was used to estimate the minimum molecular weight for the NMR-visible O ensemble. The ratio (0.58 ± 0.27) of translational diffusion coefficients (*D*) of the O (measured at the center of the O peak at −84.9 ppm) and T species (Fig. S8*C*) was converted to changes in hydrodynamic radius of O compared to that of T (13), assuming a spherical shape for O and T. This analysis indicates that O contains at least 30 protomers, with molecular weight > 400 kDa. The linewidth of the ^19^F resonance of O is linearly correlated with the O concentration (Fig. S8*B*), suggesting that exchange among the polydisperse O species may contribute to line broadening.

### Characterization of the pH 4.4 monomeric aggregation intermediate

The monomeric intermediate M is the key species connecting the native tetramer and the oligomeric species on the aggregation pathway (7). To extend the type and number of probes available for characterization of M, we recorded ^1^H,^15^N-heteronuclear single quantum coherence (HSQC) spectra of WT TTR as the pH was lowered from neutral to 4.4, where the tetramer T dissociates into M, which subsequently aggregates (8). To slow aggregation, the spectra were acquired at 277 K in the absence of salt. Under these conditions, little increase in turbidity at 330 nm was observed for 100 μM WT TTR over the course of 1 week (Fig. S9*A*). Inclusion of KCl in the buffer accelerates TTR aggregation, even at 10 μM concentration (Fig. S9*B*).

**Figure 6:**
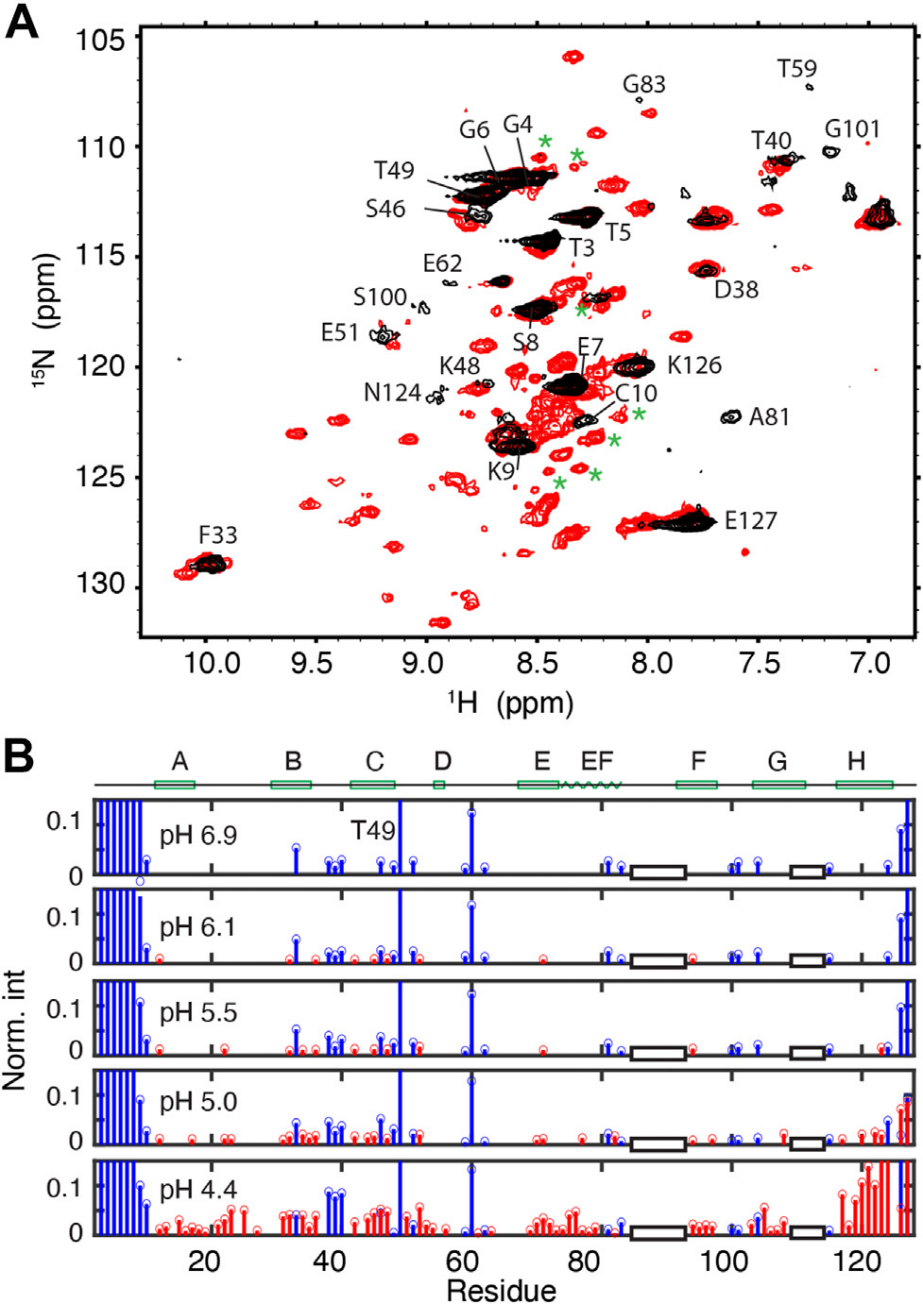
**pH Titration of WT TTR at 277 K.***A*, ^1^H,^15^N-HSQC spectral overlay of backbone amides in WT TTR at 277 K without KCl, at pH 6.9 (*black*) and 4.4 (*red*). New cross-peaks that appear due to pH-induced unfolding of WT TTR are labeled with *green asterisks*. *B*, pH titration of WT TTR from pH 6.9 to pH 4.4 at 277 K. Residues observed at pH 6.9 are shown in *blue* and additional residues that appear at lower pHs are in *red*. Cross-peak intensities (measured by peak height) at each pH value were normalized using the average intensity of residues T3, G4, and T5 in the unstructured N-terminal region. The *y*-axis scale was reduced to focus on low intensity peaks at low pHs. As a result, intensities of the N-terminal T3 to S8, T49, T60, and the C-terminal K126 and E127 are larger than the y limit. The two regions for which assignments are missing for F87 E are shown as two empty *black boxes*. TTR, transthyretin.

At neutral pH, WT TTR is predominantly a tetramer. At 277 K, the tetramer tumbles sufficiently slowly that many resonances in the ^1^H-^15^N HSQC spectrum of nondeuterated WT TTR at pH 6.9 are broadened beyond detection and only ∼28 cross-peaks are observed (Fig. 6*A*, *black*). Except for F33, S46, and K48, these cross-peaks arise from the N- or C-terminal regions or from flexible loops. As the pH is lowered, the tetramer dissociates and monomer cross-peaks appear in the spectrum. An additional ∼59 cross-peaks were observed at pH 4.4 (Fig. 6*A*, red). The gradual increase in monomer cross-peak intensity as the pH is lowered is illustrated in Figure 6*B*. Although WT TTR at 277 K and pH 4.4 in the absence of KCl is expected to populate T (∼35%), M (∼23%), and NMR-visible oligomer O (∼42%), based on ^19^F spectra of S85-TTR^F^ under the same conditions (Fig. S10), most amide cross-peaks from T and O are broad and are not observable in the HSQC spectrum under these conditions.

**Figure 7:**
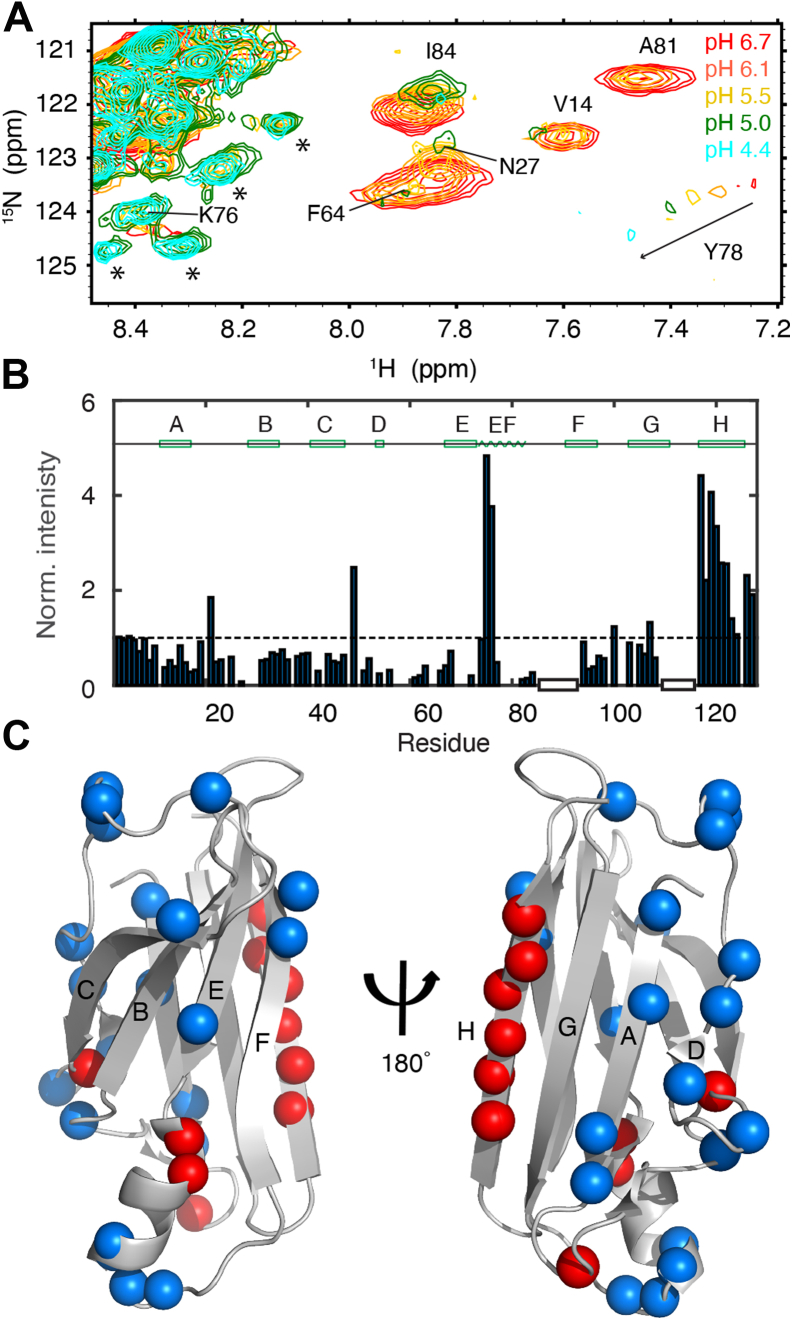
**HSQC pH titration of F87E TTR at 277 K.***A*, region of ^1^H,^15^N-HSQC spectrum of F87E at 277 K showing changes over the range pH 6.7 to pH 4.4. New cross-peaks that appear due to pH-induced unfolding of the F87E monomer are labeled with *asterisks*. *B*, normalized peak intensities of F87E at pH 4.4 relative to those at pH 6.7. An averaged intensity of the T3, G4, and T5 cross-peaks was used as reference for normalization. The *dashed line* denotes no changes in intensity. Two regions where assignments are missing are indicated by empty *black* boxes. The secondary structure of the TTR tetramer is shown at the top of the panel. *C*, structure of the TTR protomer (PDB: 5CN3) showing the backbone N atoms for residues that lose >60% intensity at pH 4.4 relative to pH 6.7 (*blue*) and whose intensity increased more than twofold (*red*). HSQC, ^1^H,^15^N-heteronuclear single quantum coherence; PDB, Protein Data Bank; TTR, transthyretin.

The changes observed in the ^1^H-^15^N HSQC spectrum of WT TTR as the pH is decreased reflect primarily the T → M transition. In order to examine the changes in the structure of M itself, we turned to F87E mutant TTR, which is a monomer at 100 μM concentration at neutral pH (Fig. S2*B*). The new cross-peaks that appear in the pH 4.4 spectrum of WT TTR overlap with those from F87E under the same conditions (Fig. S11*A*), confirming that they are indeed associated with the monomer. To assign the amide cross-peaks in the WT M spectrum, the backbone assignments of monomeric F87E (BMRB 51171) at neutral pH and 298K were first transferred to the pH 4.4 spectrum at 277 K by performing temperature and pH titrations (Figs. 7*A* and S11*B*). The resulting assignments were then transferred to the pH 4.4 spectrum of WT TTR (Fig. S11*A*). Changes in chemical shift between pH 6.7 and pH 4.4 are small (Fig. S12), with the weighted average difference in ^1^H,^15^N amide shifts less than 0.05 ppm for most residues, showing that the overall structure of the monomer is retained over this pH range.

Excluding residues that are close to titratable sidechains (His, Asp, and Glu), the only residues that exhibit larger than average shift changes are located in the EF helix and loop and in the A and G strands (Fig. S12). Of note, the cross-peak of the Y78 amide from the EF helix shows substantial pH-dependent chemical shift changes (Fig. 7*A*). Lowering the pH broadens the cross-peaks of most residues (Fig. 7*B*). Cross-peaks that lose more than 60% of their intensities relative to the pH 6.9 spectrum are associated with residues in the A-strand, the DE loop, the C-terminal residues of the EF helix, and the Schellman C-capping motif (14) in the N terminus of the EF loop (Fig. 7*C*). By contrast, cross-peaks of residues S117 to V122, which make up a large part of the H-strand, become more intense at pH 4.4 (Fig. 7*B*), suggesting that this region becomes more flexible. Several new cross-peaks appear at pH 5.0 or below in the spectra of both the F87E monomer (*black asterisks* in Figs. 7*A* and S11*A*) and WT TTR (*green asterisks* in Fig. 6*A*). The ^1^H resonances of these new cross-peaks are poorly dispersed and appear in the central random coil region (15), indicating that the corresponding residues are probably in unfolded regions.

### Real-time ^1^H,^15^N-HSQC measurements of aggregation kinetics

**Figure 8:**
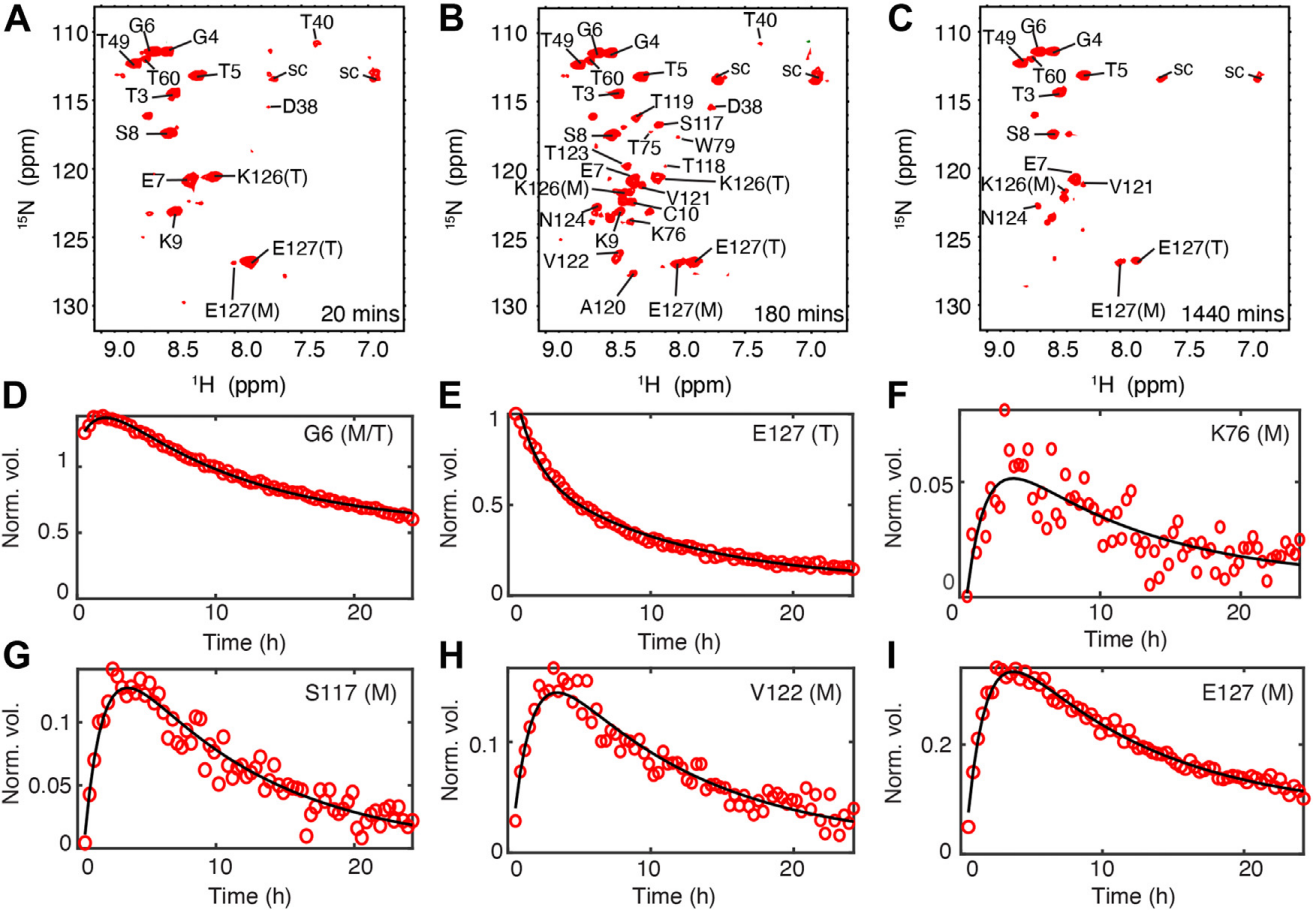
**Aggregation of 200 μM WT TTR followed by backbone amides in time-dependent**^**1**^**H,**^**15**^**N-HSQC experiments at pH 4.4 and 277 K with 100 mM KCl.***A*–*C*, HSQC spectra at the first, intermediate (with the most peaks), and last time points. Assigned cross-peaks are labeled. For clarity, sidechain NH cross-peaks in negative contours are only shown in the full spectrum in Fig. S13. *D*–*I*, time-dependent changes in backbone amide cross-peak volumes for G6 (identical chemical shifts in T and M), K76, S117, V122, E127 monomer resonances, and the tetramer E127 resonance. The cross-peak volume in (*E*) at the first time point is used for normalization. The *black lines* are fits based on a three-state aggregation kinetics model with relaxation rate constants γ_1_=0.74 ± 0.02 h^−1^ and γ_2_=0.10 ± 0.01 h^−1^. See Fig. S14 for the global fits of all resolved residues. HSQC, ^1^H,^15^N-heteronuclear single quantum coherence; TTR, transthyretin.

Aggregation of WT TTR was initiated by reducing the pH from pH 7.0 to 4.4 in the presence of 100 mM KCl and time-dependent changes in cross-peak volume were followed for 24 h (Fig. 8, *A*–*C* and S13). Three types of behavior were observed for amide cross-peaks (Fig. 8, *D*–*I*). The resonance of residue G6 (Fig. 8*D*), which is located in the unstructured N-terminal region of the TTR tetramer, is intense in the first measurement (20 min after lowering the pH) and continues to increase in intensity for another 1 to 2 h before slowly decaying. Several other residues from the disordered N-terminal region show the same behavior (Fig. S14). The transient increase in peak volume is due to the formation of M, which for these residues has similar chemical shifts as in T. The chemical shifts of these N-terminal residues are close to random coil values predicted by the POTENCI server (16) (Fig. S15). For cross-peaks that are uniquely associated with T (*e.g.*, the E127 tetramer cross-peak in Fig. 8*E*), the intensity decays steadily with time and no transient increase is observed. For resonances observed only in the HSQC spectra of M (*e.g.*, the K76, S117, V122, and E127 monomer cross-peaks in Fig. 8, *F*–*I*), the peak volumes at the first time point (20 min) are weak, reach a maximum after ∼3 to 4 h as the concentration of M builds up, and then slowly decay over 24 h as aggregation progresses. The maximum number of resolvable cross-peaks (∼45) was observed ∼3 to 4 h after lowering the pH (Figs. 8*B* and S13). As aggregation progresses, most cross-peaks in the spectrum of M disappear into noise; the remaining backbone amide cross-peaks are from the N- and C-terminal regions and show poor ^1^H chemical shift dispersion, suggesting that these regions experience high local flexibility and structural disorder in M (Fig. 8*C*).

The biphasic kinetics in Figures 5, *F*–*I* and S14 suggest a two-step, three-state model as a maximum parsimony approach for 45 relatively high signal/noise (S/N) amide cross-peaks. The time-dependent volume changes of all the observed resonances can be fitted by two global rate constants (γ_1_ = 0.74 ± 0.02 h^−1^ and γ_2_ = 0.10 ± 0.01 h^−1^, Fig. S14). The kinetic traces are best fit by a reversible T⇋M⇋A mechanism; reversibility in both steps is required for adequate fits (see Fig. S16 for examples of fits to alternative models for residues K9 and V122), consistent with the kinetic reversibility needed to fit our earlier ^19^F TTR aggregation data (7).

### Reversal of aggregation

**Figure 9:**
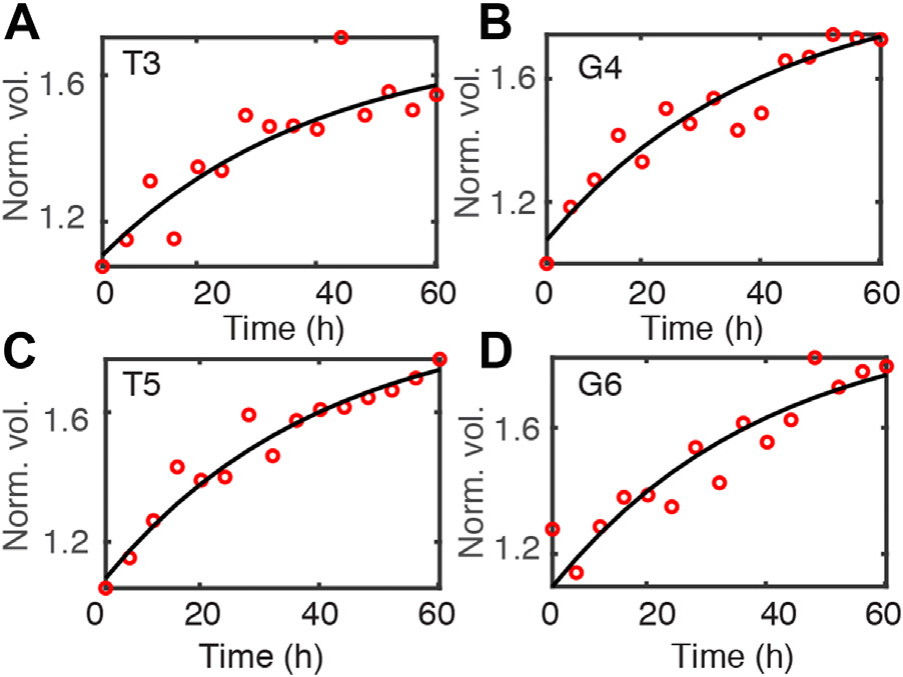
**Monitoring disaggregation of WT TTR at pH 7.0 and 277 K by**^**1**^**H,**^**15**^**N-HSQC experiments.***A*–*D*, volumes of the four observable N-terminal backbone amide cross-peaks increase as a function of time after jumping the pH from 4.4 to 7.0. The volume of the G4 amide cross-peak at time zero was used for normalization. The *black lines* are exponential fits with a global relaxation rate constant (0.03 ± 0.01 h^−1^). HSQC, 1H,15N-heteronuclear single quantum coherence; TTR, transthyretin.

Since low pH drives the coupled dissociation–aggregation equilibria of TTR forward (7), we tested whether the equilibria could be reversed by increasing the pH. ^1^H,^15^N-HSQC spectra recorded at 277 K after rapid pH jump from 4.4 to 7.0 in the presence of 100 mM KCl show an increase in the cross-peak volumes of four N-terminal residues (T3, G4, T5, and G6, Fig. 9) at a globally fitted rate constant of 0.03 ± 0.01 h^−1^. This rate constant describes the pH-driven process where M is released from an ensemble of soluble aggregates and subsequently rapidly reassembles into T at neutral pH. The rate-limiting step, likely the dissolution of soluble aggregates, is estimated by ^19^F NMR to have a rate constant of 0.02 ± 0.01 h^−1^ for S85-TTR^F^ (7), consistent with the apparent overall reverse rate constant determined here.

## Discussion

### Concerted and reversible aggregation kinetics independent of probe locations

Building on our previous work on measuring TTR aggregation using the ^19^F-BTFA probe coupled to TTR at S85C, we have extended the aggregation kinetic measurements to three additional BTFA coupling sites and to 45 amide cross-peaks in ^1^H,^15^N-HSQC spectra. For S85-TTR^F^ and S100-TTR^F^, ^19^F resonances from both the T and M species can be observed in the NMR spectrum, (Fig. 2); similarly, the ^1^H,^15^N-HSQC spectra show resonances for both T and M for the two C-terminal residues K126 and E127 (Figs. 8*B* and S13). The time series of aggregation kinetics of these peaks reveal rich kinetic insights into the early steps in TTR aggregation. More importantly, regardless of their locations within the TTR structure, the aggregation kinetics reported by both the ^19^F and amide ^15^N and ^1^H probes are the same (Figs. 3 and 8, *D*–*I*, S14), consistent with a highly concerted aggregation process during the early stages of the TTR aggregation pathway.

The global relaxation rate constants determined from time-dependent changes in the ^1^H,^15^N-HSQC cross-peak volumes in spectra of 200 μM WT TTR tetramer at 277 K/pH 4.4 (γ_1_ = 0.74 ± 0.02 and γ_2_ = 0.10 ± 0.01 h^−1^) are greater than those for 10 μM S85-TTR^F^ at the same temperature and pH (γ_1_ = 0.51 ± 0.01 and γ_2_ = 0.04 ± 0.01 h^−1^ (7)), consistent with faster TTR aggregation at higher concentrations (6). Lim *et al.* also showed that at a low concentration (12 μM) and pH 4.4, aggregation of TTR is greatly slowed such that dispersed cross-peaks can be observed in the ^1^H,^15^N-HSQC spectrum (17). Our aggregation kinetics for TTR at high concentration, measured by HSQC NMR experiments, are similar to reported values under comparable conditions: the half-time (*t*_*1/2*_) for tetramer decay determined from intensity changes of the E127 cross-peak is ∼4 h for 200 μM WT TTR at 277 K/pH 4.4 (Fig. 8*E*), comparable to the *t*_*1/2*_ of ∼3 h determined for 270 μM WT TTR at 277 K/pH 3.0 by small-angle X-ray scattering (18).

Our kinetic data also show that the early steps on the low pH TTR aggregation pathway, involving the soluble species T, M, and NMR-visible O, are fully reversible (Figs. 9, S16 and S17). Reversible aggregation of TTR has also been observed by cycling between high and low hydrostatic pressure (19). TTR is similar to other aggregation-prone proteins in undergoing reversible oligomerization (20). For example, the β2-microglobulin fibril undergoes pH-induced depolymerization to form monomers and oligomers (21), reversible oligomerization has been shown as key to quantitatively model the amyloid-β aggregation pathway (22), and aggregation intermediates of the prion protein are in dynamic equilibria (23).

### Dynamic exchange between M and soluble oligomers

The finding that M exchanges with large, NMR-invisible TTR oligomers at 298 K and pH 4.4 (Fig. 5*B*) is consistent with a small-angle X-ray scattering study, which showed that TTR monomers exchange with protofibrils of ∼2900 kDa molecular weight in acetic acid solution at 277 K and pH 3.0 (18). Analogous observations have been made for amyloid-β, where transient binding of monomer to high molecular weight protofibrils and fibrils has been quantified using NMR dark-state exchange saturation transfer (24, 25) and visualized by cryo-EM as a multistep secondary nucleation phenomenon (26).

**Figure 10:**
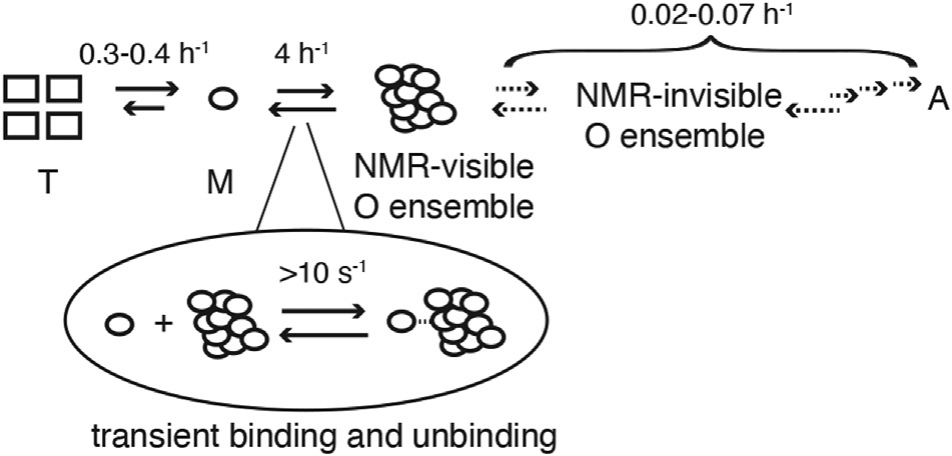
**A mechanistic scheme of TTR aggregation.** While the formation and growth of large aggregates is slow on the hour timescale, transient interaction between M and NMR-visible O is much faster on the second timescale. Rate constants were measured or modeled at 277 K and pH 4.4 using S85-TTR^F^. TTR, transthyretin.

TTR aggregation is slowed at 277 K and pH 4.4, leading to accumulation of NMR-visible oligomers that contain more than 30 protomers (>400 kDa), consistent with previous reports of 44-mer oligomers (27). Loss of overall ^19^F-NMR signal also occurs, indicating the formation of large, NMR-invisible aggregates (7). While exchange between M and large NMR-invisible aggregates or protofibrils may contribute to the increased linewidth and enhanced *R*_2_ relaxation of M at 277 K/pH 4.4, the positive linear correlation between ^19^F linewidth or Δ*R*_2_ and the concentration of the polydispersed NMR-visible oligomers (Fig. 5*C*) suggests that exchange between M and O is the dominant contribution to line broadening. At 298 K and pH 4.4, the population of NMR-visible oligomers is very low and the small Δ*R*_2_ observed for M under these conditions is thus attributed to exchange with open sites on larger, NMR-invisible aggregates. Simulations using the Bloch–McConnell equations show that the forward rate constant $\left(k_{\text{on}}^{\text{app}}\right)$ for binding of M to oligomers or aggregates must be on the order of tens per second at 277 K and pH 4.4 in order to account for the experimentally observed values of Δ*R*_2_ (Fig. S7*F*). The rate of exchange between monomer and oligomer observed by NMR relaxation is therefore orders of magnitude faster than the slow, reversible conversion between M and NMR-visible O at 277 K and pH 4.4 (∼4 h^−1^ for 10 μM S85-TTR^F^ Fig. S17). The exchange process that contributes to concentration-dependent line broadening of M involves transient association and dissociation of monomer from the growing oligomers and likely constitutes an early step in the slow assembly of large insoluble aggregates or fibrils (7). These vastly different yet concurrent kinetic processes underline the complexity of multistep protein aggregation processes with hierarchies of timescales (28) (Fig. 10).

### Site-specific conformational changes in M and NMR-visible O

Despite the concerted global aggregation kinetics observed for TTR with all of the ^19^F and ^1^H,^15^N amide probes, location-dependent conformational changes were identified in the aggregation-prone M and O intermediates. Consistent with earlier work (29, 30), cross-peaks in the HSQC spectra of WT TTR at pH 4.4 are well dispersed and overlap with those of the F87E monomer at the same pH in the absence of KCl (Fig. S11*A*), showing that the WT monomer largely retains its folded structure under these conditions. Most amide cross-peaks in spectra of F87E undergo only small chemical shift changes as the pH is lowered from 6.7 to 4.4 (Fig. S12). Biochemical evidence indicates partial unfolding of the TTR monomer at pH 4.4 (31, 32), likely enhancing structural flexibility in local regions. An X-ray structure of the WT TTR tetramer at pH 4.0 shows a major conformational rearrangement of the entire EF region in one subunit (33). However, with the exception of the amide cross-peaks of E72, D74, and Y78, the chemical shifts of residues in the EF region of F87E and M-TTR (F87M/L110M) exhibit only small changes at acidic pH (Fig. S12 and Ref. (34)), suggesting that ground state conformational changes of M in solution are much smaller than observed in the low pH X-ray structure. In agreement with previous studies of monomeric M-TTR (34), there are substantial changes in F87E cross-peak intensity over the pH range 6.7 to 4.4 (Fig. 7), consistent with increased flexibility and exchange between a native-like ground state structure and an alternative conformational state with enhanced aggregation propensity (34). At pH 4.4, the intensity of the T75 and K76 amide cross-peaks is greatly increased, suggesting enhanced dynamics in the N-terminal region of the EF helix at acidic pH. The cross-peaks of residues A81–I84, which form a Schellman C-capping motif that stabilizes the EF helix (14), become severely broadened with decreasing pH (Fig. 7), most likely due to exchange with an alternative or locally unfolded conformation of the EF loop. The EF region plays a critical role in stabilization of both the tetramer and the folded protomer (14). Mutations that disrupt packing of the EF helix onto the hydrophobic core or destabilize its helical structure facilitate tetramer dissociation and monomer unfolding and lead to increased TTR aggregation propensity (14, 35).

In the NMR-visible oligomers, the ^19^F chemical shift of the S85C-BTFA probe is upfield shifted relative to T but is downfield shifted relative to M (Fig. 4), showing that the CF_3_ group is partly solvent exposed in the oligomer but more buried than in the monomer. The S85C-BTFA probe is located in the EF loop and the change of ^19^F chemical shift indicates conformational perturbation in this region in the NMR-visible oligomeric states. The structural changes in the EF region on progressing from the tetramer to the low pH monomer and the NMR-visible oligomer, species that are involved in early steps of the aggregation pathway, are likely to predispose TTR for further conformational rearrangements in higher order aggregates. It has been shown that the EF helix becomes disordered in insoluble TTR aggregates formed at low pH *in vitro* (29, 36, 37) and that it rearranges into β strands in fibrillar amyloids extracted from cardiac tissue (38) and from the vitreous body of the eye (39) of patients carrying a V30M mutation. Interestingly, S85 is partly solvent exposed in cryo-EM structures of V30M TTR fibrils (38, 39), consistent with the solvent accessibility inferred from the ^19^F chemical shift of the S85C-BTFA probe in the NMR-visible O species.

Under aggregating conditions at pH 4.4, residues S117 to V122, which are located in the H strand of the TTR tetramer, show greatly increased amide cross-peak intensities in HSQC spectra of the WT and F87E monomers (Figs. 6*B* and 7*B*), with chemical shifts close to random coil (Fig. S15*A*). The amide ^1^H resonances of A120, V121, and V122 of F87E have large chemical shift temperature coefficients (Fig. S18), suggesting that these residues do not participate in hydrogen bonded β-sheet structure in the TTR monomer, even at neutral pH. Indeed, the H-strand was observed to be unfolded in NMR solution structures of M-TTR at 500 bar and pH 6.5 (40). The increase in cross-peak intensity at acidic pH (Figs. 6 and 7) is consistent with enhanced conformational fluctuations under conditions that promote aggregation. Fluctuations of the H-strand that lead to breaking of intramolecular hydrogen bonds would potentially expose highly amyloidogenic segments of the G β strand (38) and promote entry into the aggregation pathway. Antiserum against the 115 to 124 segment of TTR reacts with the TTR amyloid in ATTRwt patients but not with the native TTR in plasma or in pancreatic islet alpha cells (41, 42). It is likely that the H-strand, which is well folded and protected in the native T state, is partly solvent exposed in the monomer and in higher order TTR aggregates and fibrils.

Finally, residues from the D-strand and DE loop in M, including E63, show decreased amide cross-peak intensities in HSQC spectra of F87E under aggregation conditions (Fig. 7, *B* and *C*), indicating increased exchange broadening at acidic pH. ^19^F NMR data obtained with the E63C-BTFA probe show that it is less solvent exposed in the NMR-visible oligomers than in the tetramer (Fig. 4). Interestingly, E63 is partly buried in the cryo-EM structures of V30M TTR cardiac fibrils (38) and the vitreous fibrils (39), and both fibrillar structures show distinct local packing of the DE loop residues against neighboring β strands.

In conclusion, NMR experiments using ^19^F BTFA and amide ^15^N and ^1^H probes have revealed the concerted aggregation kinetics of TTR and location-dependent conformational changes in the TTR tetramer (T), monomer (M), and NMR-visible oligomer (O). M transiently and reversibly exchanges with polydisperse TTR species, including NMR-visible and NMR-invisible oligomers. Early steps in the TTR aggregation pathway involving T, M, and the heterogeneous oligomers are highly reversible. The solvent accessibility of ^19^F probes located in the DE and EF loops in the NMR-visible O species also follows a comparable trend as in the V30M *ex vivo* fibrillar structures (38, 39). These observations suggest that certain location-dependent structural features in early steps of TTR aggregation could propagate to later stages *via* aggregation intermediates, likely including M and NMR-visible O. Future experiments to explore how these conformational changes are coupled to TTR amyloid formation as a function of mutation are clearly warranted.

## Experimental procedures

### Protein expression, purification, and labeling

A pET29 plasmid encoding a C10S TTR sequence with an N-terminal Met residue was used as the cloning template (7). Site-directed mutagenesis of S46C and E63C was introduced to the template by the QuikChange Kit (Agilent) using the previously reported primer sequences (10). The mutation S100C was introduced using the polymerase incomplete primer extension as an updated protocol (43). The forward and reverse primer sequences of S100C were:

5′-CGACTGCGGCCCCCGCCGCTAC-3′

5′-GGCCGCAGTCGTTGGCTGTGAATACCAC-3′.

The proteins were expressed in the *Escherichia coli* BL21(DE3) Star strain as previously described (35).

The purification of protonated WT TTR and the monomeric F87E mutant was carried out according to a published two-step protocol (35), where a gel filtration separation by Sephacryl S100 was followed by an anion exchange step using a Capto Q ImpRes column. For C10S-S46C, C10S-E63C, and C10S-S100C, any disulfide-bridged TTR oligomers formed during protein expression were reduced by 10 mM tris(2-carboxyethyl)phosphine at 298 K for 1 to 3 h and reloaded to the Capto Q column to remove high molecular weight impurities (7). The purified TTR protein was exchanged into 10 mM potassium phosphate buffer at pH 7.0 with 100 mM potassium chloride (NMR buffer). A molar extinction coefficient of 18,450 M^−1^ cm^−1^ was used to calculate the monomer/protomer concentration of TTR, which is used throughout this work. The BTFA labeling was carried out as previously described (7). Briefly, 2 mM BTFA was mixed with tris(2-carboxyethyl)phosphine–reduced TTR mutants (∼100 μM) at 298 K for 1 h in NMR buffer, prior to separation using a PD25 desalting column equilibrated in NMR buffer.

### Turbidity assay

Assays were performed using 10 μM C10S-S46C-BTFA (denoted as S46-TTR^F^), C10S-E63C-BTFA (E63-TTR^F^), and C10S-S100C-BTFA (S100-TTR^F^) in 50 mM sodium acetate and 100 mM KCl at pH 4.4 (aggregation buffer) at 310 K as previously described (8, 35).

### ^19^F-NMR aggregation assay

The real-time ^19^F-NMR aggregation assays were performed as previously reported (7). Briefly, aggregation of 10 μM S46-TTR^F^, E63-TTR^F^, or S100-TTR^F^ was initialized by mixing the proteins in NMR buffer with aggregation buffer at 310 K. ^19^F NMR spectra were recorded using a Bruker Avance 600 spectrometer with a ^1^H/^19^F–^13^C/^15^N QCI cryoprobe and shielded z-gradient coil or a Bruker Avance 700 spectrometer with a ^1^H/^19^F–^13^C/^15^N TCI cryoprobe and shielded z-gradient coil. Each 1D spectrum was acquired with 4k complex points with an acquisition time of 91 or 182 ms. The carrier offset was set at −84.2 ppm. The recycle delay was set at 1 s, which is about three times longer than the ^19^F *T*_1_ relaxation time for the tetramer resonance in all ^19^F-labeled samples (Table S3). The decay of the ^19^F-NMR resonance was recorded with a time resolution of 1 h over a total of 65 experiments. Unless otherwise noted, 10% D_2_O was included as the lock signal in all NMR experiments. The 1D NMR datasets were processed using NMRPipe (44) (https://www.ibbr.umd.edu/nmrpipe) and analyzed using MATLAB.

### ^1^H,^15^N-HSQC aggregation and disaggregation assay

Aggregation of 200 μM ^15^N-labeled WT TTR in aggregation buffer at 277 K was monitored by recording real-time ^1^H,^15^N-HSQC spectra. The measurements were performed using a Bruker Avance 600 spectrometer. The spectra comprised [2k, 256] complex points in the ^1^H,^15^N dimensions with spectral widths of [16, 32] ppm. The carrier offset was set to 4.7 and 118 ppm for the ^1^H,^15^N dimensions, respectively. A recycle delay of 1 s was used. For each delay in the indirect dimension (*t*_1_), four scans were collected, giving rise to a total acquisition time of 20 min per 2D spectrum. A total of 72 spectra were recorded. The 2D NMR datasets were processed using NMRPipe (44) and analyzed using Sparky (45).

In the real-time HSQC disaggregation assay, 175 μl of the aggregated TTR sample from the aforementioned aggregation experiment was added to 70 μl of 200 mM dibasic potassium phosphate buffer containing 100 mM KCl to give a final pH of 7.0 after mixing. Only the cross-peaks of the first four N-terminal residues (T3, G4, T5, and G6) were visible above the noise in the initial HSQC spectrum of the aggregated protein. Disaggregation at 277 K was monitored by recording a series of HSQC spectra with the same parameters as those in the forward aggregation assay, except that 12 scans (1 h) were acquired for each *t*_1_ delay to increase the S/N ratio. A total of 60 spectra were collected.

### NMR aggregation data analysis

For 1D ^19^F-NMR data processing, a 1 Hz exponential line-broadening factor was applied to the free induction decay, which was then zero-filled to 16k before Fourier transformation. The populations of the various species involved were quantified using peak areas. The missing ^19^F signal amplitude was fitted to a single exponential function to determine aggregation rate constants. For mutants with resolvable T and M ^19^F resonances, a three-state fit (T⇋M⇋A) was performed as previously described (7). The concentrations of NMR-visible O and M were determined by fitting ^19^F spectra using three Lorentzian functions and compared to the total peak areas at *t* = 0.

In the ^1^H,^15^N-HSQC forward aggregation analysis, a Lorentz–Gaussian window function was employed in the direct time domain and a squared sine-bell window function with an offset of 0.45 π was used in the indirect dimension. The peak volumes were extracted using the box sum method in CcpNmr (46). A global fit to a three-state model (T⇋M⇋A) was performed and two global relaxation rate constants (γ_1_ and γ_2_) were obtained. In addition, two amplitudes and one offset were fitted for each resonance. The uncertainties associated with the global fit were calculated as one SD (68% confidence). Resonance assignments were transferred from the published backbone amide assignment of WT TTR (47) and F87E (BMRB accession numbers 27514 and 51171) by temperature and pH titration as described later.

NMR data for the ^1^H,^15^N-HSQC disaggregation assay were processed as described in the previous paragraph. To enhance the S/N ratio, data from four successive time points were summed. The peak volumes of T3, G4, T5, and G6 were used for analysis, where a global single exponential relaxation rate constant and the amplitude/offset for each residue were determined.

### NMR titrations for the monomeric F87E and tetrameric WT TTR

^1^H,^15^N-HSQC spectra of F87E at 140 μM in 50 mM Bis–Tris–4-morpholineethansulfonic acid (MES) buffer at pH 6.7 were recorded at 298, 291, 284, and 277 K using a Bruker Avance 600 spectrometer. A pH titration was performed at 277 K by gradually adding 50 mM MES to the F87E sample at pH 6.7 to lower the pH. To reduce aggregation, the concentration of F87E was gradually lowered (90 μM at pH 6.1, 60 μM at pH 5.5, 50 μM at pH 5.0, and 40 μM at pH 4.4). Similar protocols were applied for the WT TTR titration, where 100 μM WT TTR in 50 mM Bi–Tris–MES buffer at pH 6.9 and 277 K was gradually diluted while the pH was lowered (100 μM at pH 6.1, 80 μM at pH 5.5, 70 μM at pH 5.0, and 50 μM at pH 4.4). KCl was omitted from the buffer so that TTR aggregation was slowed to facilitate NMR measurements (48).

### ^19^F-NMR DOSY (^19^F-DOSY)

Formation of M on the aggregation pathway of S100-TTR^F^ was verified by ^19^F-NMR DOSY measurements (7, 49). The DOSY experiment was performed on a 600 MHZ spectrometer using 50 μM S100-TTR^F^ at pH 4.4 and 298 K in aggregation buffer from which KCl was omitted. To enhance the S/N, 5000 scans were acquired for each of 10 evenly spaced relative z-gradient strengths ranging from 5% to 50%, resulting in a total acquisition time of 18 h. The data were analyzed using the Stejskal–Tanner equation (50) and the slopes for the T and M species were compared. The fitting uncertainty was determined by one SD of 50 bootstrapped datasets (51). To estimate the molecular weight range of the NMR-visible oligomers (O), the ^19^F-DOSY data for O at 277 K/pH 4.4 with a total concentration of 80 μM S85-TTR^F^ were analyzed using the Stokes–Einstein equation as described in Ref (7).

### ^19^F *R*_2_ relaxation

^19^F *R*_2_ relaxation rate constants were measured using a Carr–Purcell–Meiboom–Gill pulse sequence (52). Data were collected in a Bruker Avance 600 spectrometer for samples shortly after a pseudoequilibrium population of TTR species was reached by incubation for periods ranging from overnight to 1 to 2 days, before precipitation occurred (>1 week). Interleaved sets of 180^o^ ^19^F pulses (with 2, 4, 8, 16, 32, 64, 128, 256, or 512 pulses in the train) were applied and the delay between successive 180^o^ pulses was set to be 250 μs (corresponding to a fast-pulsing frequency of 4000 s^−1^). The recycling delay was set to 2 s giving a typical run time of ∼10 to 20 h. The ^19^F spectrum with the highest S/N was fitted to an appropriate number of Lorentzian peaks to extract peak centers. The ^19^F *R*_2_ was then determined by fitting the decays of intensities at these peak centers using single exponential functions.

### Numerical simulations of the Bloch–McConnell equation to estimate ^19^F Δ*R*_2_

The ^19^F Δ*R*_2_ (transverse relaxation rate constant *R*_2_ in the presence of exchange broadening, minus *R*_2_ without exchange) of M can be described by the homogeneous form of the Bloch–McConnell equation as previously shown (11, 12). The ^19^F relaxation was modeled assuming two-state exchange (F⇄B) between free (F) and bound (B) states of M. The pseudo first-order rate constant $k_{\text{on}}^{\text{app}}=k_{\text{on}}{\left[\text{O}\right]}_{\text{free}}$ describes the forward reaction, where ${\left[\text{O}\right]}_{\text{free}}$ is the concentration of available monomer binding sites in oligomers and $k_{\text{on}}$ is the second-order association rate constant; the first-order rate constant $k_{\text{off}}$ describes the reverse reaction.

Simulation parameters were set to experimentally measured values for monomeric TTR at 277 K/pH 4.4 ($R_{1}^{\text{F}}=3.0\;{\text{s}}^{-1},\;R_{2}^{\text{F}}=33.7{\text{ s}}^{-1}$, peak position of M = −85.0 ppm). The *R*_1_ value (2.4 s^−1^) of the NMR-visible oligomers in 80 μM S85-TTR^F^ at 277 K/pH 4.4 was used to approximate $R_{1}^{\text{B}}$. The peak position of the B state was set as −84.9 ppm for the NMR-visible oligomers. The peak positions of the F and B states and the ^19^F carrier frequency (−84.2 ppm) were converted to rad/s using the frequency of ^19^F (564.9 × 2π × 10^6^ rad/s) on the 600 MHz spectrometer. The time-dependent evolution of the magnetization vector, which was set to be completely transverse initially, was carried out using the *expm* function in MATLAB and delays of 10 and 30 milliseconds were used to calculate ^19^F Δ*R*_2_ for M per Ref (12).

## Data availability

All data are reported in this paper and its supporting information.

## Supporting information

This article contains supporting information (3 tables and 18 figures) (7, 10, 16, 35, 53, 54, 55, 56).

## Conflict of interest

The authors declare that they have no conflicts of interest with the contents of this article.

## Supporting information

TTR_JBC_SI_revised

## References

[1] Johnson S.M., Connelly S., Fearns C., Powers E.T., Kelly J.W. (2012). The transthyretin amyloidoses: from delineating the molecular mechanism of aggregation linked to pathology to a regulatory-agency-approved drug. J. Mol. Biol..

[2] Eisele Y.S., Monteiro C., Fearns C., Encalada S.E., Wiseman R.L., Powers E.T. (2015). Targeting protein aggregation for the treatment of degenerative diseases. Nat. Rev. Drug Discov..

[3] Pitkänen P., Westermark P., Cornwell G.G. (1984). Senile systemic amyloidosis. Am. J. Path..

[4] Connors L.H., Lim A., Prokaeva T., Roskens V.A., Costello C.E. (2003). Tabulation of human transthyretin (TTR) variants, 2003. Amyloid.

[5] Sekijima Y., Wiseman R.L., Matteson J., Hammarström P., Miller S.R., Sawkar A.R. (2005). The biological and chemical basis for tissue-selective amyloid disease. Cell.

[6] Hurshman A.R., White J.T., Powers E.T., Kelly J.W. (2004). Transthyretin aggregation under partially denaturing conditions is a downhill polymerization. Biochemistry.

[7] Sun X., Dyson H.J., Wright P.E. (2018). Kinetic analysis of the multistep aggregation pathway of human transthyretin. Proc. Natl. Acad. Sci. U. S. A..

[8] Lai Z., Colon W., Kelly J.W. (1996). The acid-mediated denaturation pathway of transthyretin yields a conformational intermediate that can self-assemble into amyloid. Biochemistry.

[9] Ye L., Larda S.T., Frank Li Y.F., Manglik A., Prosser R.S. (2015). A comparison of chemical shift sensitivity of trifluoromethyl tags: optimizing resolution in ^19^F NMR studies of proteins. J. Biomol. NMR.

[10] Sun X., Jaeger M., Kelly J.W., Dyson H.J., Wright P.E. (2018). Mispacking of the Phe87 side chain reduces the kinetic stability of human transthyretin. Biochemistry.

[11] Helgstrand M., Härd T., Allard P. (2000). Simulations of NMR pulse sequences during equilibrium and non-equilibrium chemical exchange. J. Biomol. NMR.

[12] Libich D.S., Fawzi N.L., Ying J., Clore G.M. (2013). Probing the transient dark state of substrate binding to GroEL by relaxation-based solution NMR. Proc. Natl. Acad. Sci. U. S. A..

[13] Cavanagh J., Fairbrother W.J., Palmer A.G., Rance M., Skelton N.J. (2007).

[14] Ferguson J.A., Sun X., Dyson H.J., Wright P.E. (2021). Thermodynamic stability and aggregation kinetics of EF helix and EF loop variants of transthyretin. Biochemistry.

[15] Yao J., Dyson H.J., Wright P.E. (1997). Chemical shift dispersion and secondary structure prediction in unfolded and partly folded proteins. FEBS Lett..

[16] Nielsen J.T., Mulder F.A.A. (2018). POTENCI: prediction of temperature, neighbor and pH-corrected chemical shifts for intrinsically disordered proteins. J. Biomol. NMR.

[17] Lim K.H., Dasari A.K.R., Hung I., Gan Z., Kelly J.W., Wemmer D.E. (2016). Structural changes associated with transthyretin misfolding and amyloid formation revealed by solution and solid-state NMR. Biochemistry.

[18] Groenning M., Campos R.I., Hirschberg D., Hammarström P., Vestergaard B. (2015). Considerably unfolded transthyretin monomers preceed and exchange with dynamically structured amyloid protofibrils. Sci. Rep..

[19] Foguel D., Suarez M.C., Ferrao-Gonzales A.D., Porto T.C., Palmieri L., Einsiedler C.M. (2003). Dissociation of amyloid fibrils of alpha-synuclein and transthyretin by pressure reveals their reversible nature and the formation of water-excluded cavities. Proc. Natl. Acad. Sci. U. S. A..

[20] Dear A.J., Michaels T.C.T., Meisl G., Klenerman D., Wu S., Perrett S. (2020). Kinetic diversity of amyloid oligomers. Proc. Natl. Acad. Sci. U. S. A..

[21] Tipping K.W., Karamanos T.K., Jakhria T., Iadanza M.G., Goodchild S.C., Tuma R. (2015). pH-induced molecular shedding drives the formation of amyloid fibril-derived oligomers. Proc. Natl. Acad. Sci. U. S. A..

[22] Michaels T.C.T., Šarić A., Curk S., Bernfur K., Arosio P., Meisl G. (2020). Dynamics of oligomer populations formed during the aggregation of Alzheimer’s Aβ42 peptide. Nat. Chem..

[23] Larda S.T., Simonetti K., Al-Abdul-Wahid M.S., Sharpe S., Prosser R.S. (2013). Dynamic equilibria between monomeric and oligomeric misfolded states of the mammalian prion protein measured by ^19^F NMR. J. Am. Chem. Soc..

[24] Fawzi N.L., Ying J., Torchia D.A., Clore G.M. (2010). Kinetics of amyloid β monomer-to-oligomer exchange by NMR relaxation. J. Am. Chem. Soc..

[25] Fawzi N.L., Ying J., Ghirlando R., Torchia D.A., Clore G.M. (2011). Atomic-resolution dynamics on the surface of amyloid-β protofibrils probed by solution NMR. Nature.

[26] Törnquist M., Cukalevski R., Weininger U., Meisl G., Knowles T.P.J., Leiding T. (2020). Ultrastructural evidence for self-replication of Alzheimer-associated Aβ42 amyloid along the sides of fibrils. Proc. Natl. Acad. Sci. U. S. A..

[27] Kelly J.W., Colon W., Lai Z., Lashuel H.A., McCulloch J., McCutchen S.L. (1997). Transthyretin quaternary and tertiary structural changes facilitate misassembly into amyloid. Adv. Prot. Chem..

[28] Knowles T.P.J., Vendruscolo M., Dobson C.M. (2014). The amyloid state and its association with protein misfolding diseases. Nat. Rev. Mol. Cell Biol..

[29] Lim K.H., Dasari A.K.R., Hung I., Gan Z., Kelly J.W., Wright P.E. (2016). Solid-state NMR studies reveal native-like β-Sheet structures in transthyretin amyloid. Biochemistry.

[30] Dasari A.K.R., Hung I., Gan Z., Lim K.H. (2019). Two distinct aggregation pathways in transthyretin misfolding and amyloid formation. Biochim. Biophys. Acta Prot. Proteom..

[31] Jiang X., Smith C.S., Petrassi H.M., Hammarström P., White J.T., Sacchettini J.C. (2001). An engineered transthyretin monomer that is nonamyloidogenic, unless it is partially denatured. Biochemistry.

[32] Quintas A., Vaz D.C., Cardoso I., Saraiva M.J., Brito R.M. (2001). Tetramer dissociation and monomer partial unfolding precedes protofibril formation in amyloidogenic transthyretin variants. J. Biol. Chem..

[33] Palaninathan S.K., Mohamedmohaideen N.N., Snee W.C., Kelly J.W., Sacchettini J.C. (2008). Structural insight into pH-induced conformational changes within the native human transthyretin tetramer. J. Mol. Biol..

[34] Lim K.H., Dyson H.J., Kelly J.W., Wright P.E. (2013). Localized structural fluctuations promote amyloidogenic conformations in transthyretin. J. Mol. Biol..

[35] Sun X., Dyson H.J., Wright P.E. (2017). Fluorotryptophan incorporation modulates the structure and stability of transthyretin in a site-specific manner. Biochemistry.

[36] Lim K.H., Dasari A.K.R., Ma R., Hung I., Gan Z., Kelly J.W. (2017). Pathogenic mutations induce partial structural changes in the native β-sheet structure of transthyretin and accelerate aggregation. Biochemistry.

[37] Dasari A.K.R., Hung I., Michael B., Gan Z., Kelly J.W., Connors L.H. (2020). Structural characterization of cardiac ex vivo transthyretin amyloid: insight into the transthyretin mmisfolding pathway *in vivo*. Biochemistry.

[38] Schmidt M., Wiese S., Adak V., Engler J., Agarwal S., Fritz G. (2019). Cryo-EM structure of a transthyretin-derived amyloid fibril from a patient with hereditary ATTR amyloidosis. Nat. Commun..

[39] Iakovleva I., Hall M., Oelker M., Sandblad L., Anan I., Sauer-Eriksson A.E. (2021). Structural basis for transthyretin amyloid formation in vitreous body of the eye. Nat. Commun..

[40] Oroz J., Kim J.H., Chang B.J., Zweckstetter M. (2017). Mechanistic basis for the recognition of a misfolded protein by the molecular chaperone Hsp90. Nat. Struct. Mol. Biol..

[41] Gustavsson A., Engstrom U., Westermark P. (1994). Mechanisms of transthyretin amyloidogenesis. Antigenic mapping of transthyretin purified from plasma and amyloid fibrils and within *in situ* tissue localizations. Am. J. Path..

[42] Bergström J., Engström U., Yamashita T., Ando Y., Westermark P. (2006). Surface exposed epitopes and structural heterogeneity of *in vivo* formed transthyretin amyloid fibrils. Biochem. Biophys. Res. Comm..

[43] Klock H.E., Lesley S.A. (2009). The Polymerase Incomplete Primer Extension (PIPE) method applied to high-throughput cloning and site-directed mutagenesis. Meth. Mol. Biol..

[44] Delaglio F., Grzesiek S., Vuister G.W., Guang Z., Pfeifer J., Bax A. (1995). NMRPipe: a multidimensional spectral processing system based on UNIX pipes. J. Biomol. NMR.

[45] Goddard T.D., Kneller D.G. (2006).

[46] Vranken W.F., Boucher W., Stevens T.J., Fogh R.H., Pajon A., Llinas M. (2005). The CCPN data model for NMR spectroscopy: development of a software pipeline. Proteins.

[47] Leach B.I., Zhang X., Kelly J.W., Dyson H.J., Wright P.E. (2018). NMR measurements reveal the structural basis of transthyretin destabilization by pathogenic mutations. Biochemistry.

[48] Hammarström P., Jiang X., Deechongkit S., Kelly J.W. (2001). Anion shielding of electrostatic repulsions in transthyretin modulates stability and amyloidosis: insight into the chaotrope unfolding dichotomy. Biochemistry.

[49] Altieri A.S., Hinton D.P., Byrd R.A. (1995). Association of biomolecular systems *via* pulsed field gradient NMR self-diffusion measurements. J. Am. Chem. Soc..

[50] Stejskal E.O., Tanner J.E. (1965). Spin diffusion measurements: spin echoes in the presence of a time-dependent field gradient. J. Chem. Phys..

[51] Efron B. (1979). Rietz lecture—bootstrap methods—another look at the jackknife. Ann. Stat..

[52] Meiboom S., Gill D. (1958). Modified spin-echo method for measuring nuclear relaxation times. Rev. Sci. Instrum..

[53] Edwards J.M., Bramham J.E., Podmore A., Bishop S.M., van der Walle C.F., Golovanov A.P. (2019). ^19^F Dark-state exchange saturation transfer NMR reveals reversible formation of protein-specific large clusters in high-concentration protein mixtures. Anal. Chem..

[54] Bain A.D. (2003). Chemical exchange in NMR. Prog. Nucl. Magn. Res. Spec..

[55] Reeves L.W., Shaw K.N. (1970). Nuclear magnetic resonance studies of mutli-site chemical exchange. I. Matrix formulation of the Bloch equations. Can. J. Chem..

[56] Baxter N.J., Williamson M.P. (1997). Temperature dependence of ^1^H chemical shifts in proteins. J. Biomol. NMR.

