# Supplementary material for: A transthyretin monomer intermediate undergoes local unfolding and transient interaction with oligomers in a kinetically concerted aggregation pathway
Source: J Biol Chem. 2022 Jun 18;298(8):102162. doi: 10.1016/j.jbc.2022.102162 (PMC9293765; doi:10.1016/j.jbc.2022.102162)
Supplement: TTR_JBC_SI_revised [file mmc1.docx]

**Supporting information:**

**A transthyretin monomer intermediate undergoes local unfolding and transient interaction with oligomers in a kinetically concerted aggregation pathway**

Xun Sun, James A. Ferguson, H. Jane Dyson, Peter E. Wright*

**Affiliations:**

Department of Integrative Structural and Computational Biology and Skaggs Institute of Chemical Biology, The Scripps Research Institute, 10550 North Torrey Pines Road, La Jolla, California 92037, US

Correspondence to Peter E. Wright: [wright@scripps.edu](mailto:wright@scripps.edu)

## Table S1. Fitted aggregation rate constants for S100-TTR^F^ and S85-TTR^F^ at pH 4.4 and 310 K

| Constructs | *k*_1_ (h^-1^) ^a^ | *k*_-1_ (h^-1^) ^a^ | *k*_2_ (h^-1^) ^a^ | *k*_-2_ (h^-1^) ^a^ | γ_2_ rate constant (h^-1^) ^a^ |
| --- | --- | --- | --- | --- | --- |
| S100-TTR^F^ | 0.09 ± 0.04 | 0.8 ± 0.3 | 0.9 ± 0.1 | 0.01 ± 0.01 | 0.06 ± 0.01  (97%) ^c^ |
| S85-TTR^F, b^ | 0.10 ± 0.01 | 0.8 ± 0.1 | 0.73 ± 0.03 | 0.03 ± 0.01 | 0.06 ± 0.01  (96%) ^c^ |

^a^ The uncertainty was calculated as one standard deviation from 50 bootstrapped datasets. The kinetic model is

$T\leftrightharpoons M\leftrightharpoons A$, where T, M and A stand for tetramer, monomeric intermediates, and aggregates, respectively.

^b^ The kinetic parameters are from Ref (1) for comparison.

^c^ The relative weight of the slow relaxation rate constant is denoted as γ_2_ in Ref­­­ (1). This rate is comparable to the single-exponential fits for the combined T and M signals for all the four BTFA-bearing mutants and approximates the increase of the OD_330_ increase (Figure 3A).

## Table S2. ^19^F linewidth of M and T of S85-TTR^F^ and S100-TTR^F^ at pH 4.4 at 298 K and 277 K ^a^

| Constructs | Temp (K) | Linewidth of M (Hz) | Linewidth of T (Hz) |
| --- | --- | --- | --- |
| S85-TTR^F^ | 298 | 12 | 13 |
| S85-TTR^F, b^ | 277 | 22 | 24 |
| S100-TTR^F^ | 298 | 14 | 14 |
| S100-TTR^F^ | 277 | 26 | 22 |

^a^ The concentration used was 10 µM.

^b^ Based on a three-state Lorentzian line-shape fit for observed peaks of T, M and oligomers. The linewidths reported for S85-TTR^F^ at 298 K and for S100-TTR^F^ at both temperatures are from two-state line-shape fits.

## Table S3. Longitudinal relaxation time constant (*T*_1_) for tetrameric and monomeric TTR in four BTFA-labeled mutants at various solution conditions

| Constructs | Temp (K) | | pH | | *T*_1_ (s) for tetramer (T) ^a^ | *T*_1_ (s) for monomer (M) ^a^ | |
| --- | --- | --- | --- | --- | --- | --- | --- |
| S85-TTR^F^ | | 277 | | 4.4 | 0.35 ± 0.02 | 0.39 ± 0.03 |  |
| S85-TTR^F^ | | 298 | | 4.4 | 0.33 ± 0.03 | 0.39 ± 0.06 |  |
| S85-TTR^F^ | | 277 | | 7.0 | 0.33 ± 0.01 | N.A.^c^ |  |
| S85-TTR^F^ | | 298 | | 7.0 | 0.33 ± 0.01 | N.A. |  |
| S85-TTR^F^ | | 310 | | 7.0 | 0.35 ± 0.01 | N.A. |  |
| S46-TTR^F^ | | 298 | | 7.0 | 0.36 ± 0.01 | N.A. |  |
| S46-TTR^F, b^ | | 298 | | 4.4 | 0.36 ± 0.04 | 0.36 ± 0.04 |  |
| E63-TTR^F^ | | 298 | | 7.0 | 0.36 ± 0.01 | N.A. |  |
| S100-TTR^F^ | | 298 | | 7.0 | 0.35 ± 0.01 | N.A. |  |

^a^ The uncertainty was calculated as one standard deviation from 50 bootstrapped datasets.

^b^ The ^19^F resonances of T and M species were degenerate (chemical shift difference <0.01 ppm) for S46-TTR^F^ at pH 4.4.

^c^ N.A. means resonances not observed


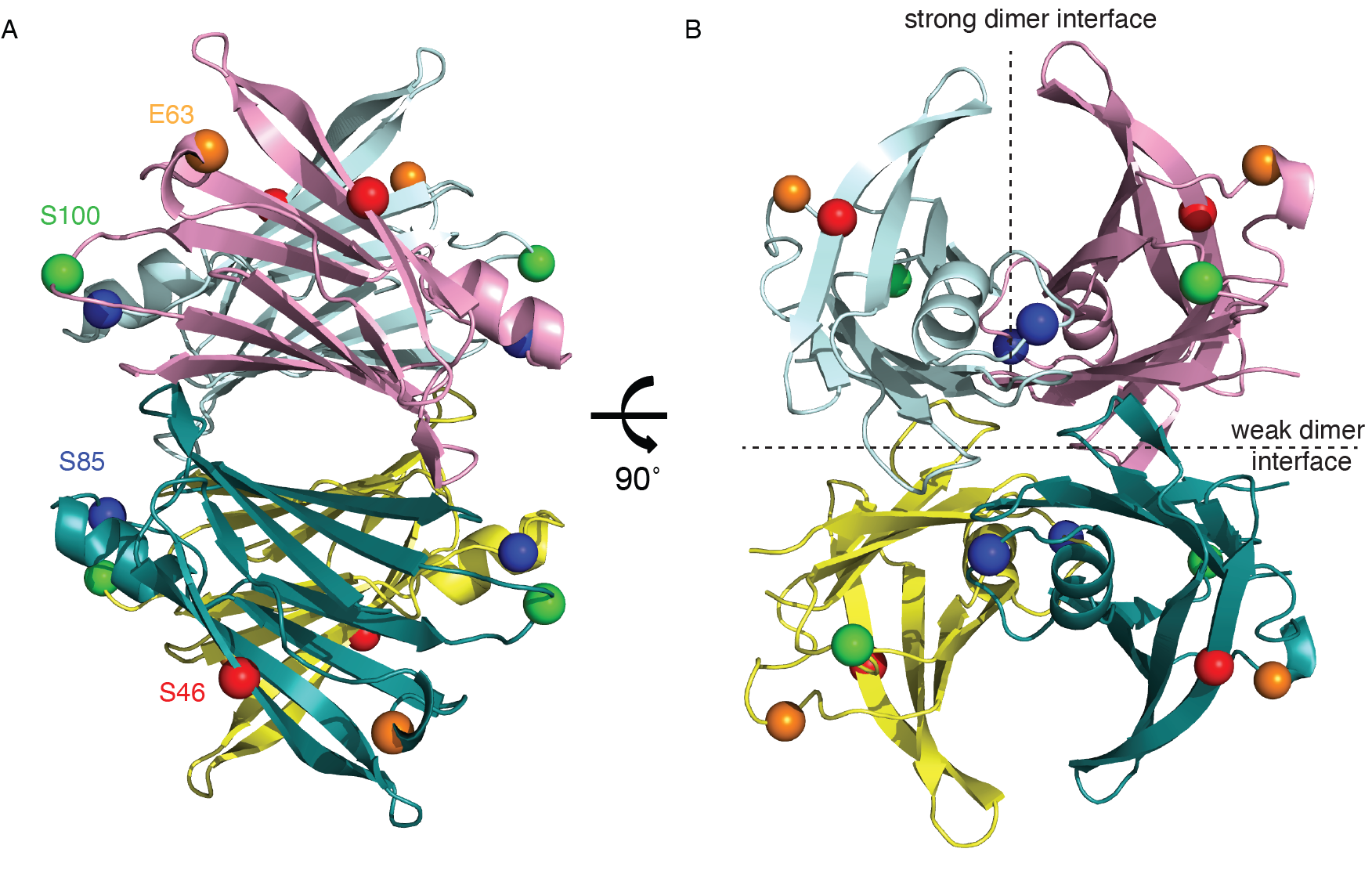


Figure S1. Locations of four ^19^F-BTFA labels on a TTR tetramer (PDB: 5CN3). Cartoon of TTR structure is colored by protomers and the Cα atoms of labeling sites are shown as color coded spheres.


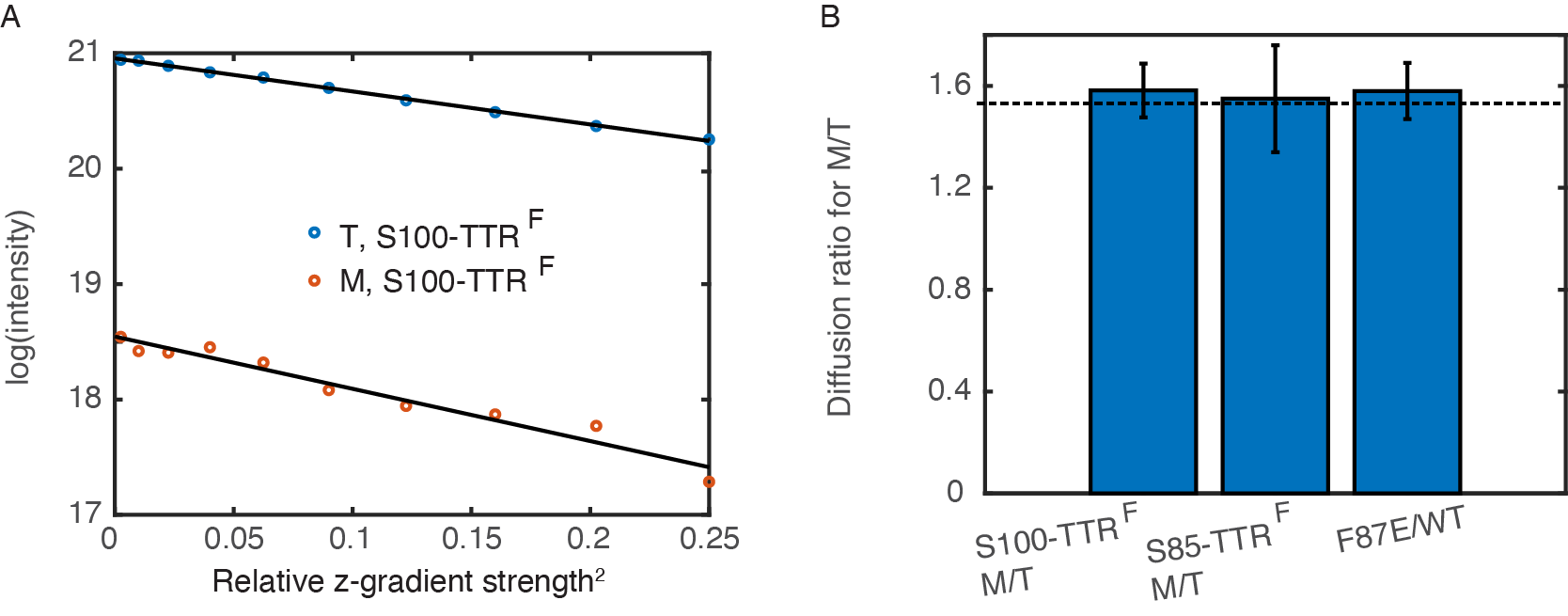


Figure S2. ^19^F-DOSY experiments to verify the M species in S100-TTR^F^. (A) ^19^F-DOSY experiment of S100-TTR^F^ at 298 K and pH 4.4 without 100 mM KCl. Black lines denote linear fits. (B) The ratios of the diffusion coefficient of M over T. The slope ratio of S100-TTR^F^ in (A) is 1.6 ± 0.1, which is consistent with the prediction using the Stokes-Einstein equation (1.53, dashed line). For comparison, diffusion coefficient ratios for S85-TTR^F^ at 277 K and pH 4.4 (Ref­­­ (1)), for F87E (monomer, 100 µM, Ref (2)), and for WT TTR (tetramer, 100 µM, Ref (3)) at 298 K and pH 7.0 are also plotted. Error bars in (B) are fitting uncertainties, estimated as one standard deviation from 50 bootstrapped datasets.


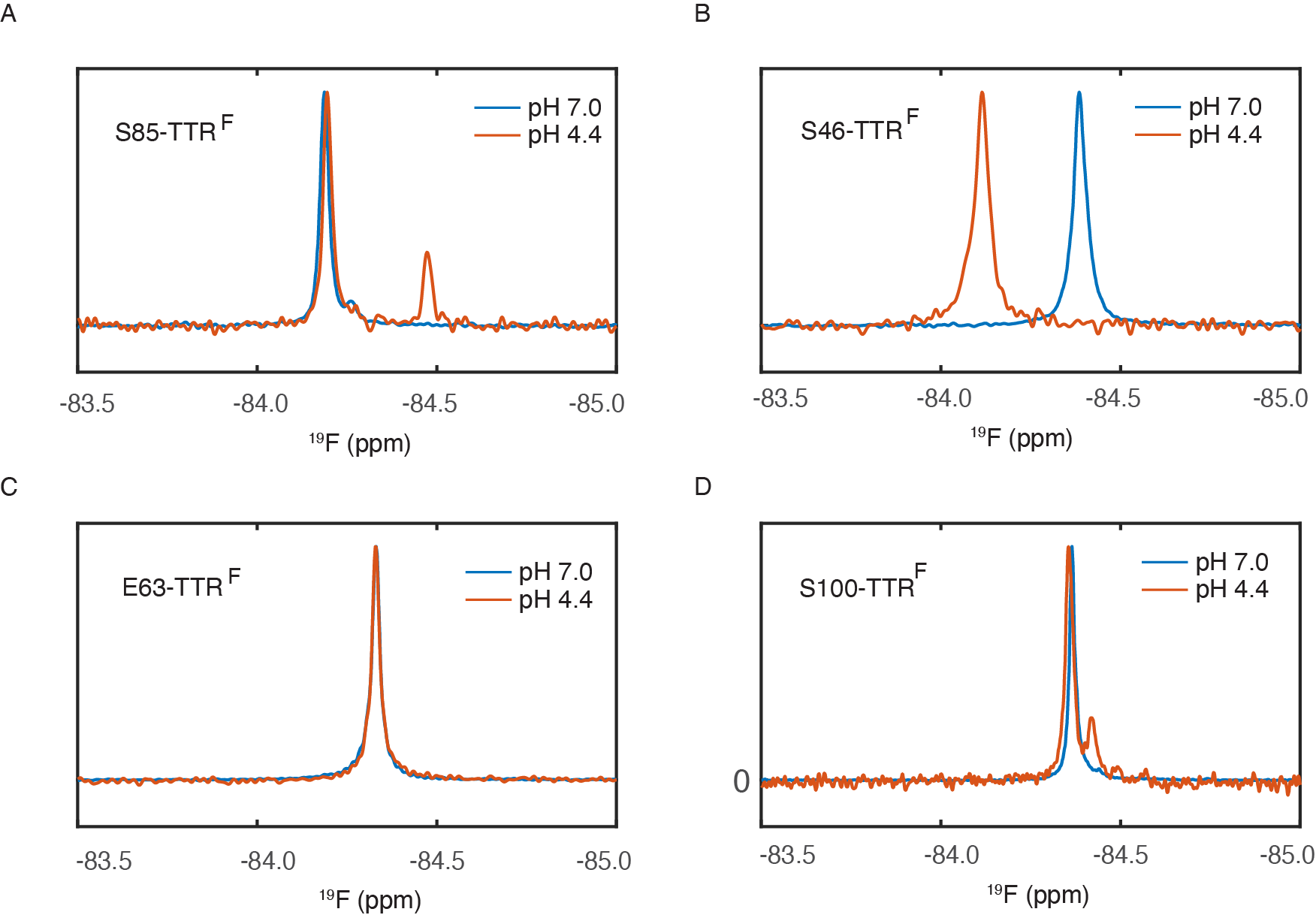


Figure S3. Comparison of ^19^F-NMR spectra of the four TTR^F^ mutants at pH 7.0/298 K and pH 4.4/298 K.


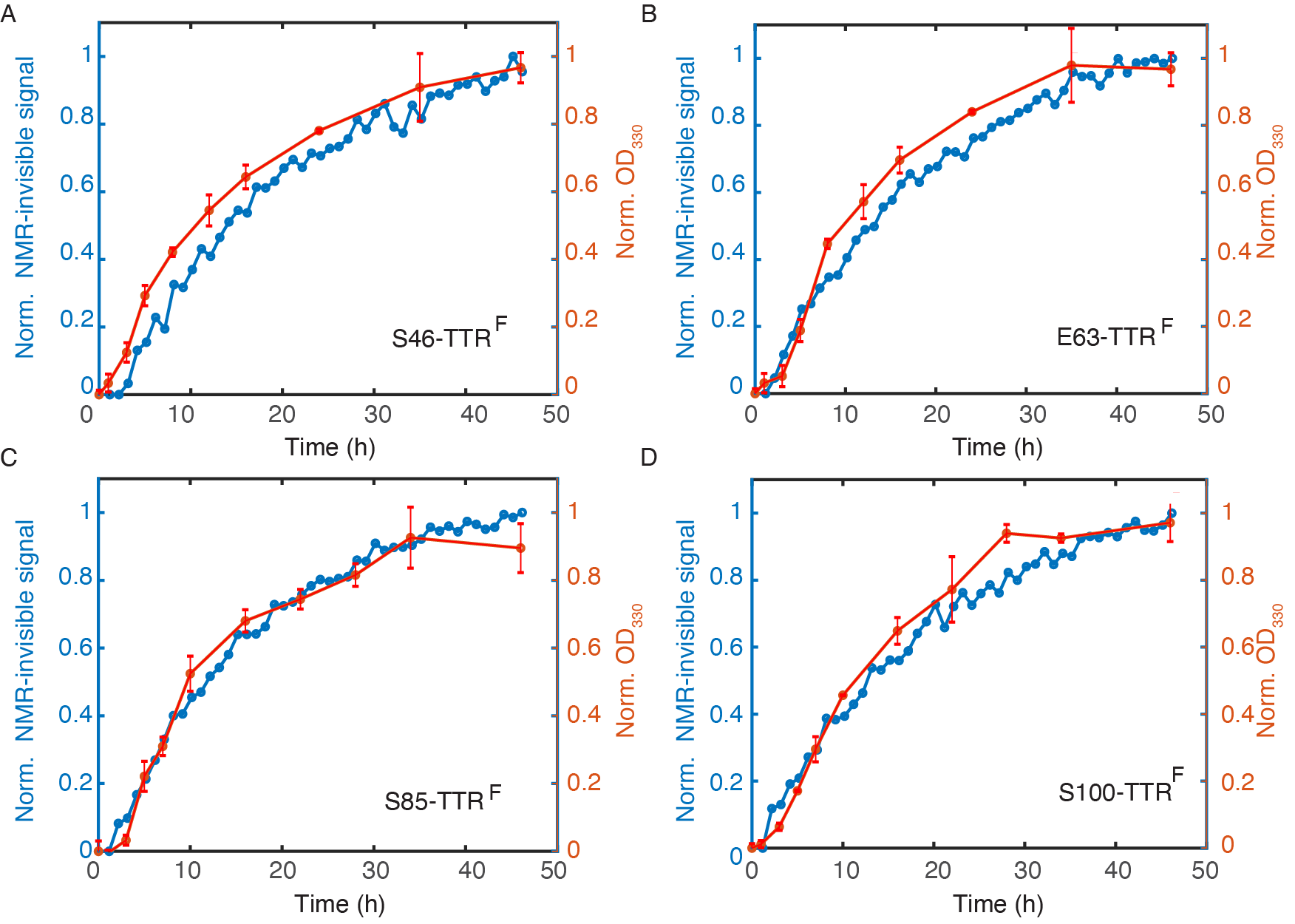


Figure S4. Comparison of NMR-invisible signal with changes in turbidity at 330 nm (OD_330_) for the four ^19^F-BTFA labeled TTR constructs (10 µM) following initiation of aggregation at pH 4.4 and 310 K. The error bars represent one standard deviation from three independent measurements. For S85-TTR^F^ and S100-TTR^F^, the signal from T and M is combined. For each mutant, the formation of ^19^F-NMR-invisible species closely mirrors the increase in OD_330_. Both axes were normalized by the maximal signals and the connecting lines are to guide the eye. The data for S85-TTR^F^ in (C) were replotted from Ref (1) for comparison.


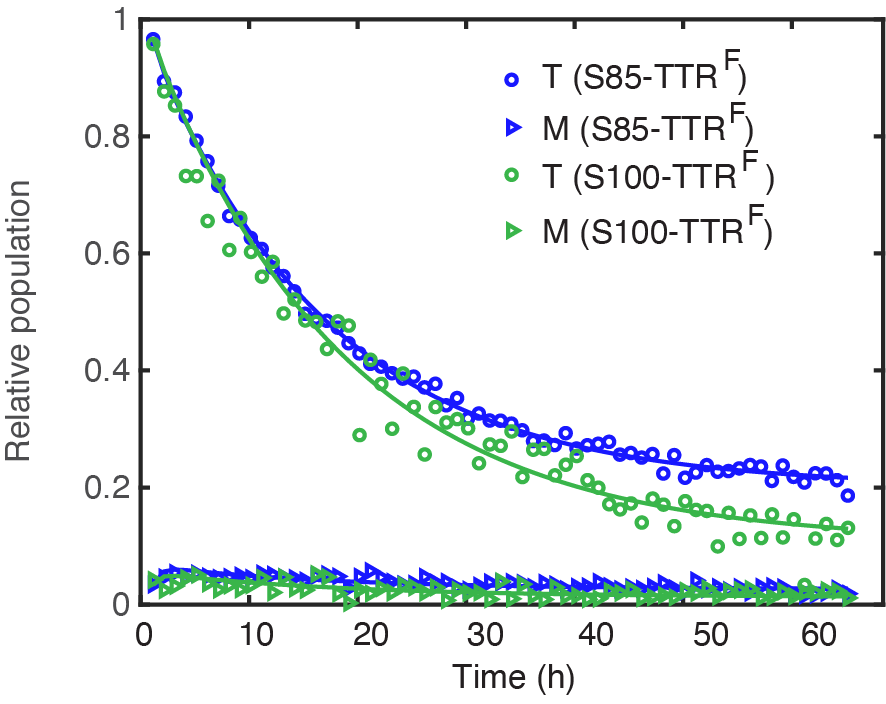


Figure S5. Similar time-dependent population changes of T and M in S85-TTR^F^ and S100-TTR^F^at pH 4.4 and 310 K. Relative populations were normalized by peak areas at *t*=0. The solid lines are fits from the three-state kinetic scheme (see Table S1 for rate constants and Figure 2B for spectral comparison). The S85-TTR^F^ data were replotted from Ref (1) for comparison.


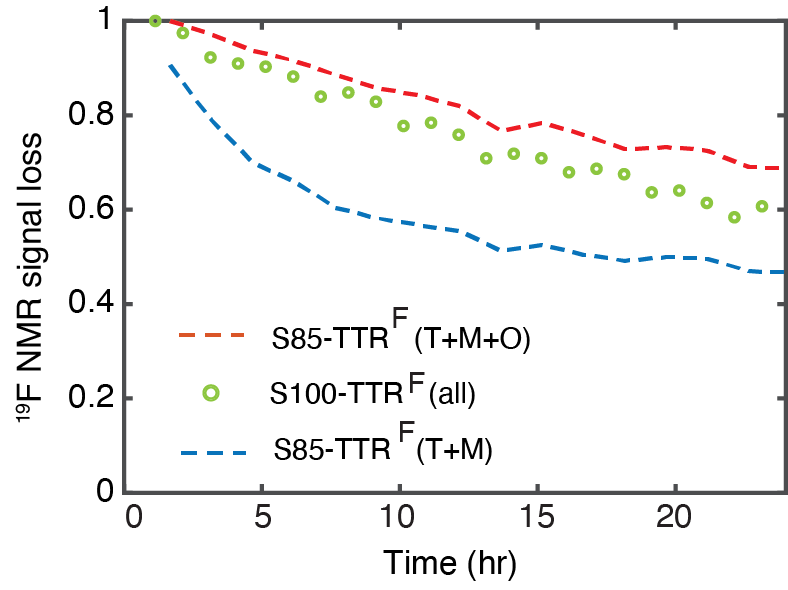


Figure S6. Time-dependent ^19^F-NMR signal loss at 277 K and pH 4.4. The two dashed lines show the signal loss for S85-TTR^F^ (blue: tetramer plus monomer, red: tetramer plus monomer plus oligomer). The overall signal loss for S100-TTR^F^ (green circle) lie between the two dashed lines, an indication that the oligomer contributes to the overall signal of the ^19^F probe in this position.


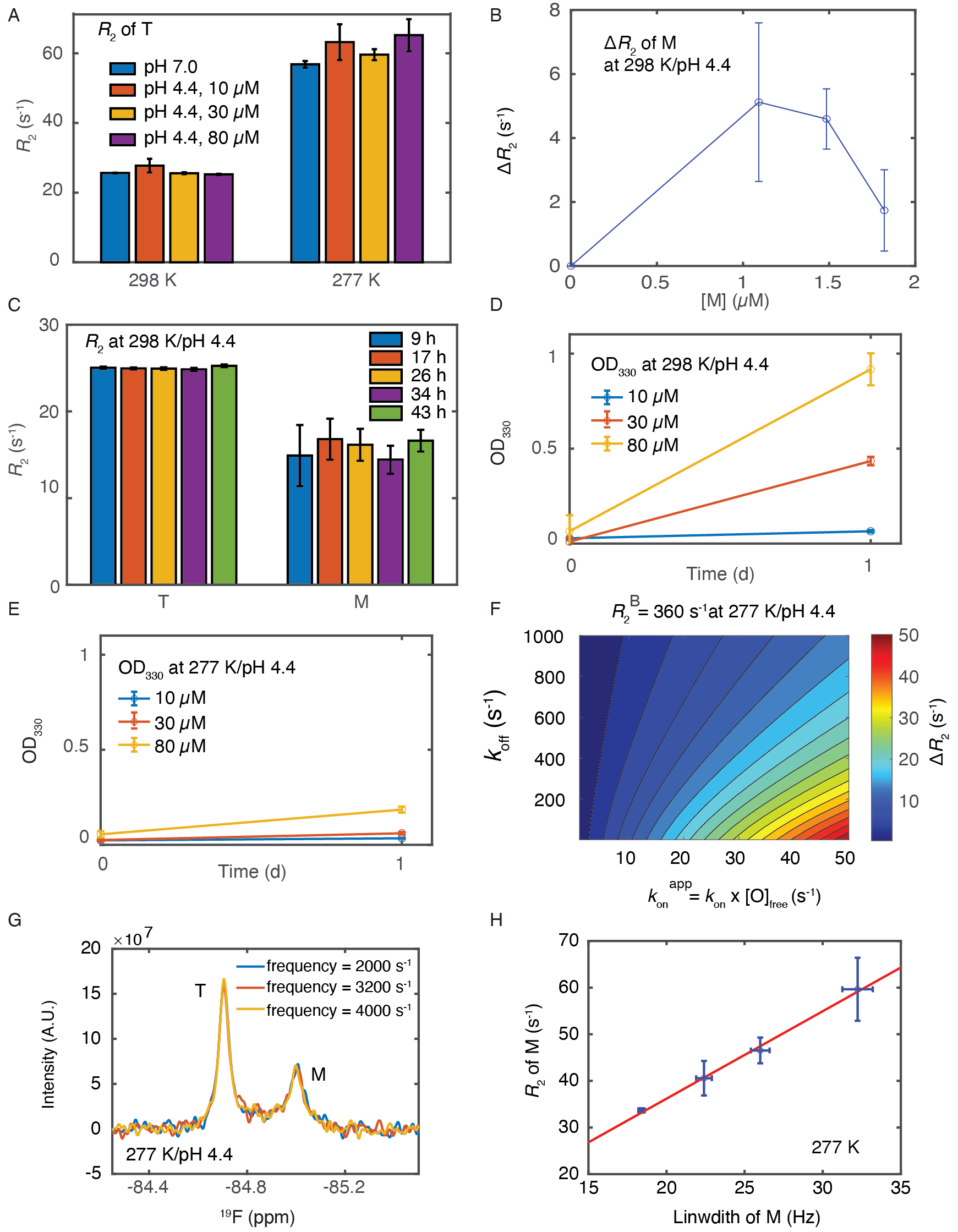


Figure S7. Experimental data and numerical simulations to study ^19^F Δ*R*_2_ of M of S85-TTR^F^. (A) Measured ^19^F *R*_2_ of T in S85-TTR^F^ at 298 K and 277 K. The *ca*. 2-fold difference in ^19^F *R*_2_ of T between the two temperatures can be accounted by changes in temperature-dependent solution viscosity according to analysis in Ref (4). (B) The lack of positive linear correlations between [M] and ^19^F Δ*R*_2_ of M at 298 K/pH 4.4. (C) Measured ^19^F *R*_2_ values of the T and M peaks of the S85-TTR^F^ spectra do not depend on the aggregation time at pH 4.4 within 9–43 h. The total TTR concentration was 80 µM and the temperature was 298 K. Error bars in panels (A) and (C) are fitting uncertainties, estimated as one standard deviation from 50 bootstrapped datasets. (D­–E) Aggregation of 10, 30 and 80 µM S85-TTR^F^ at pH 4.4 at 298 K (D) and 277 K (E) in aggregation buffer, monitored by OD_330_. (F) Simulated ^19^F Δ*R*_2_ contour plots of M as a function of $k_{\mathrm{on}}^{\mathrm{app}}$and $k_{\mathrm{off}}$ at pH 4.4/277 K with $R_{2}^{B}$ = 360 s^-1^ as the *R*_2_ values of the NMR-visible O species (measured at the center of the O peak at -84.9 ppm for 80 µM TTR at 277 K and pH 4.4). For reference, the Δ*R*_2_ experimentally observed for M, which increases from 7 s^-1^ at 10 µM to 26 s^-1^ at 80 µM. Therefore, the experimental Δ*R*_2_ values can be explained by physically reasonable $k_{\mathrm{on}}^{\mathrm{app}}$and $k_{\mathrm{off}}$ values in the two-state model in the main text. (G) ^19^F NMR spectra of 80 µM S85-TTR^F^ at 277 K/pH 4.4, measured with three pulsing frequencies in a constant-time (10 ms) CPMG experiment. The peak heights of T and M in the three spectra are similar, indicating that any conformational sampling of M on the millisecond timescale is quenched by a pulsing frequency at 4000 s^-1^ used in the ^19^F *R*_2_ measurements. (H) Linear correlation between the *R*_2_ and linewidth of M at 277 K (*R* = 0.998). The data point with the smallest *R*_2_ and linewidth is measured for the M species in the ^19^F spectrum of S85-TTR^F^-F87A at 277 K/pH 7.0. The other data points are based on the M peak in 10, 30 and 80 µM S85-TTR^F^ at 277 K/pH 4.4. The y-intercept of the linear fit is close to 0 (-1) and the slope (1.9) is less than π due to exchange (5,6).


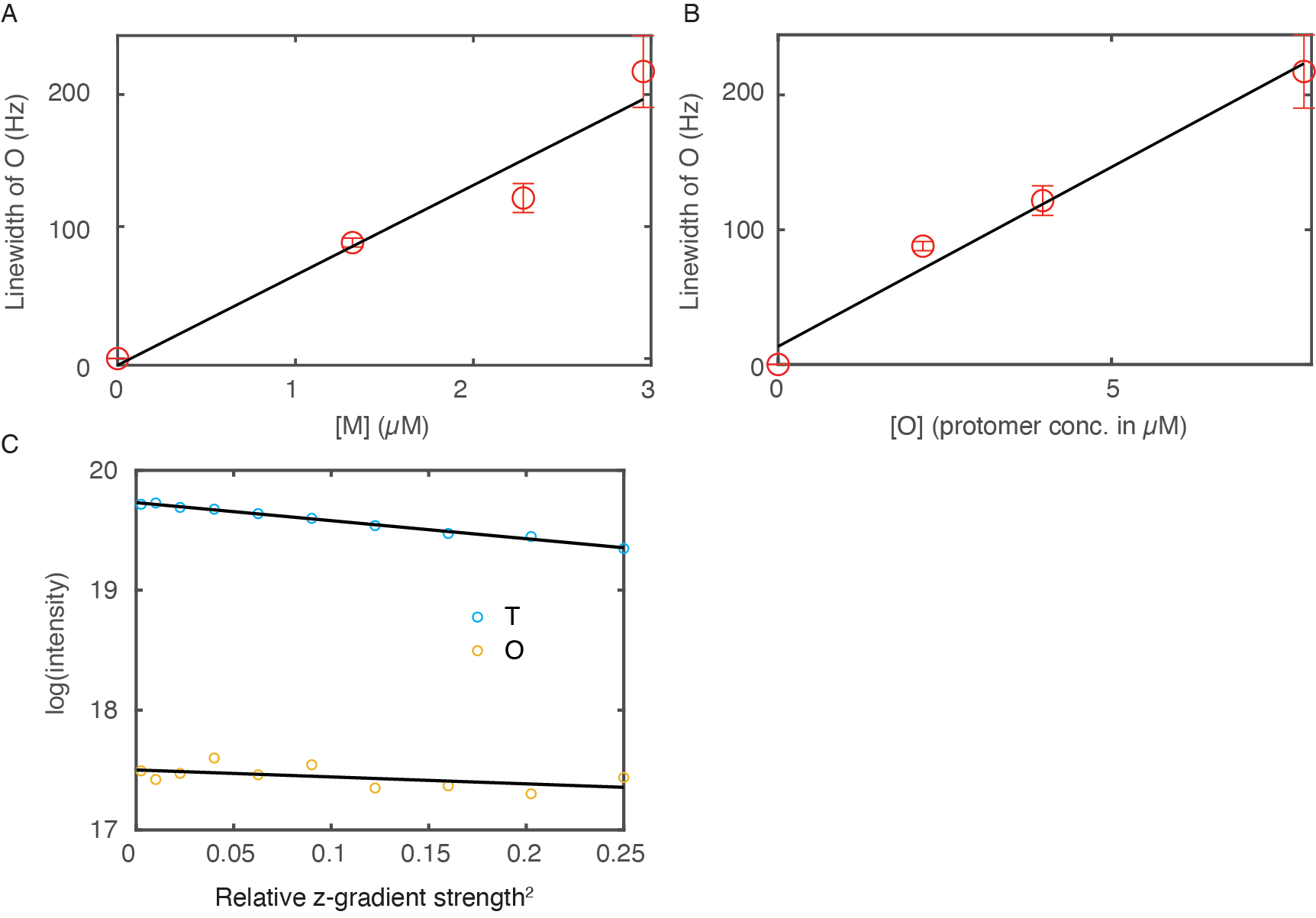


Figure S8. Study of the NMR-visible O species at 277 K/pH 4.4. (A, B) Plot of the ^19^F linewidth of the NMR-visible O species as a function of the [M] (A) or the protomer concentration of [O] (B). The black lines are linear fits to the four data points. No data are shown for the sample at 200 µM total TTR concentration because the O peak is too broad to allow reliable estimation of the linewidth. (C) ^19^F-DOSY experiment for 80 µM S85-TTR^F^ at 277 K/ pH 4.4. The black lines are linear fits for the T and NMR-visible O species and the ratio of the slope (O/T) is 0.58 ± 0.27. The peak height at the center of the O peaks was used in quantification.


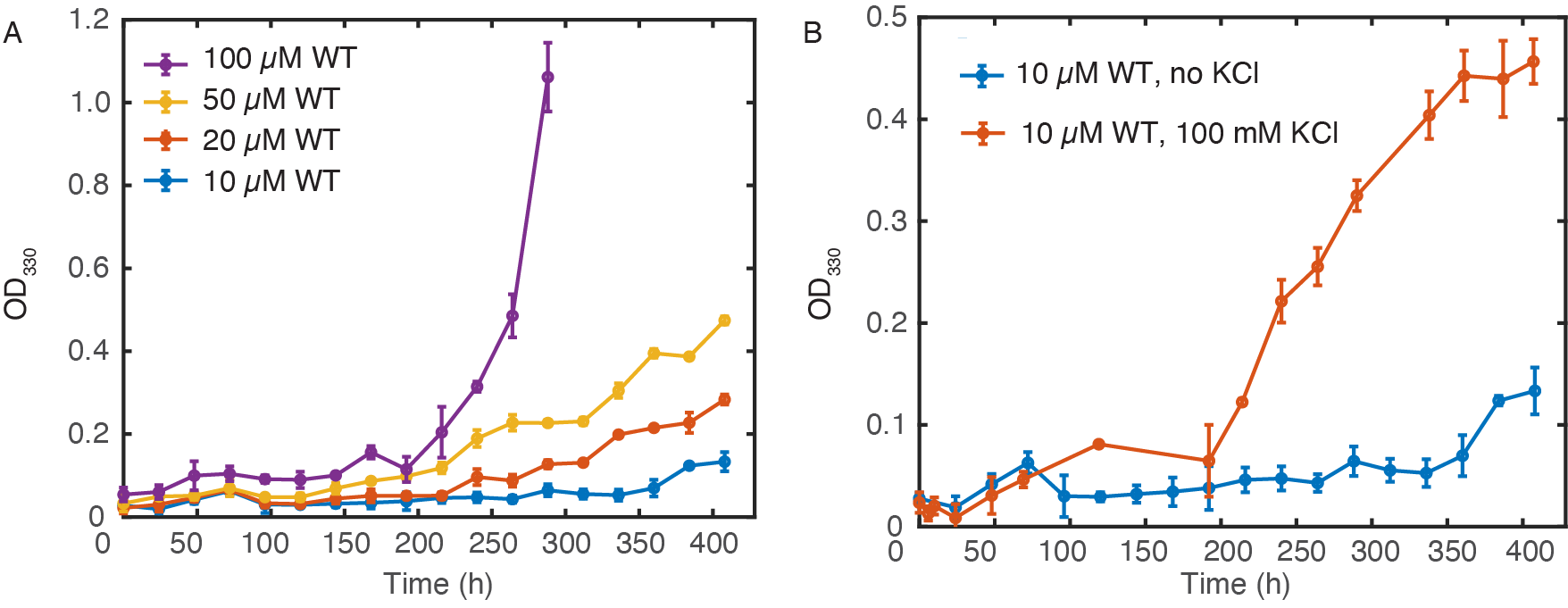


Figure S9. Aggregation of WT TTR. (A) WT TTR of different concentrations at pH 4.4 and 277 K without KCl. (B) Time course of aggregation of 10 µM WT TTR with and without 100 mM KCl.


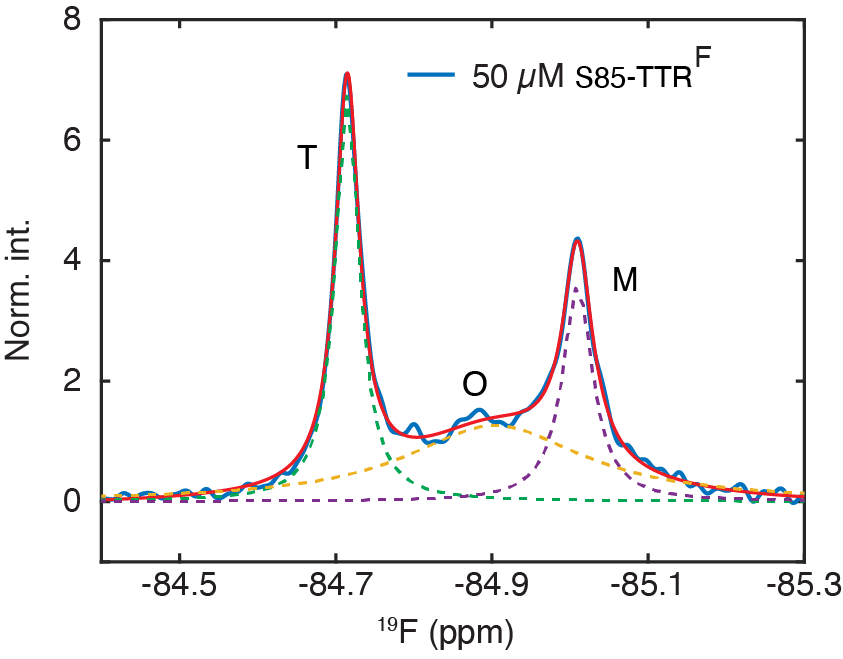


Figure S10. The deconvoluted ^19^F-NMR spectrum of 50 µM S85-TTR^F^ at pH 4.4 and 277 K without KCl. The dashed lines denote three Lorentzian fits (green for T, purple for M and orange for O). The summed population from three Lorentzian is shown in red. The population is 35% for T, 23% for M and 42% for O.


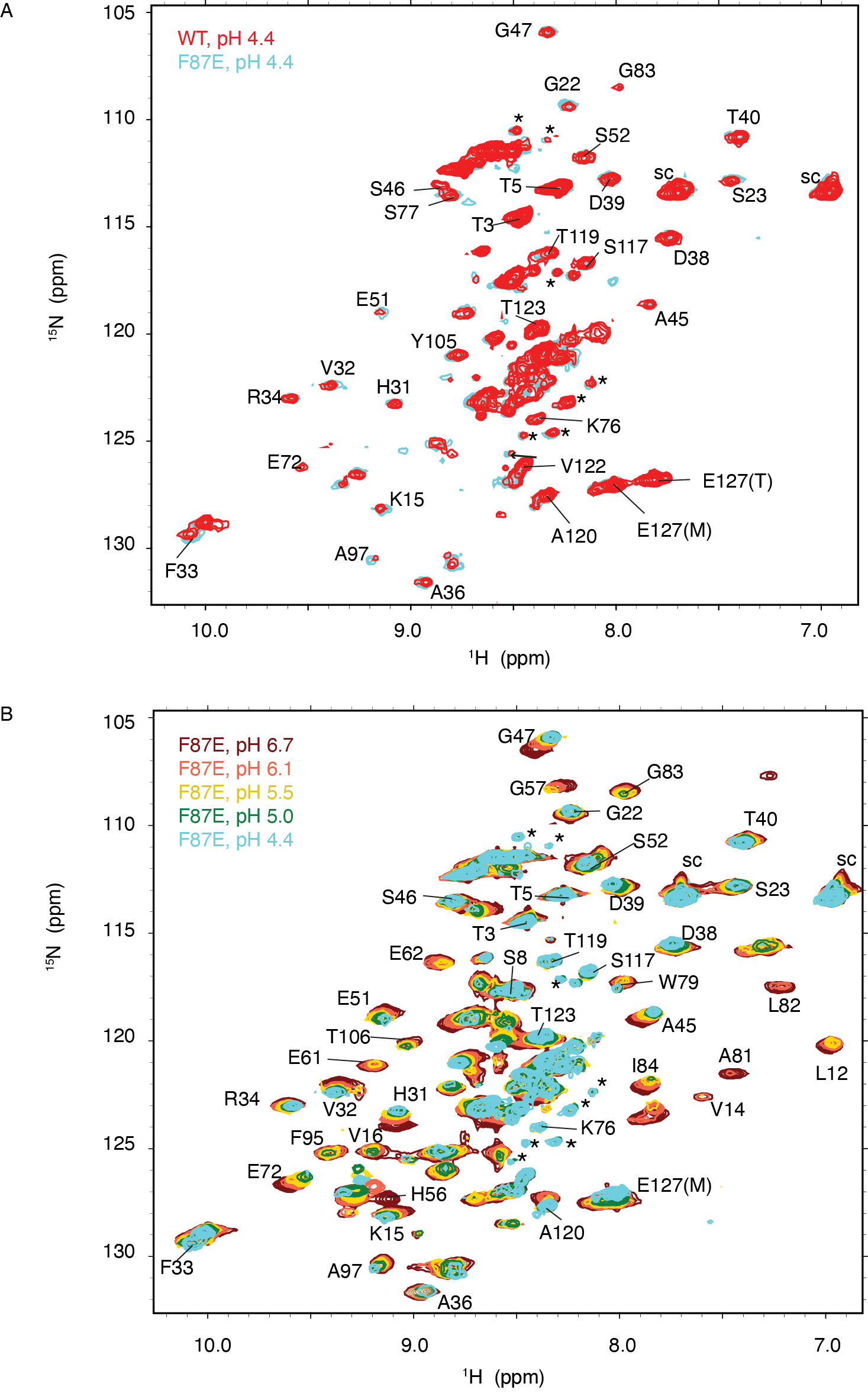


Figure S11. Full ^1^H,^15^N-HSQC spectra. (A) Spectral c­­­omparison of WT (red) and F87E (cyan) at pH 4.4 and 277 K. (B) pH titration of F87E at 277 K. New cross peaks that arise from pH-induced unfolding of the F87E monomer are labeled with black asterisks in both (A) and (B). Sidechain NH cross peaks are labeled as sc.


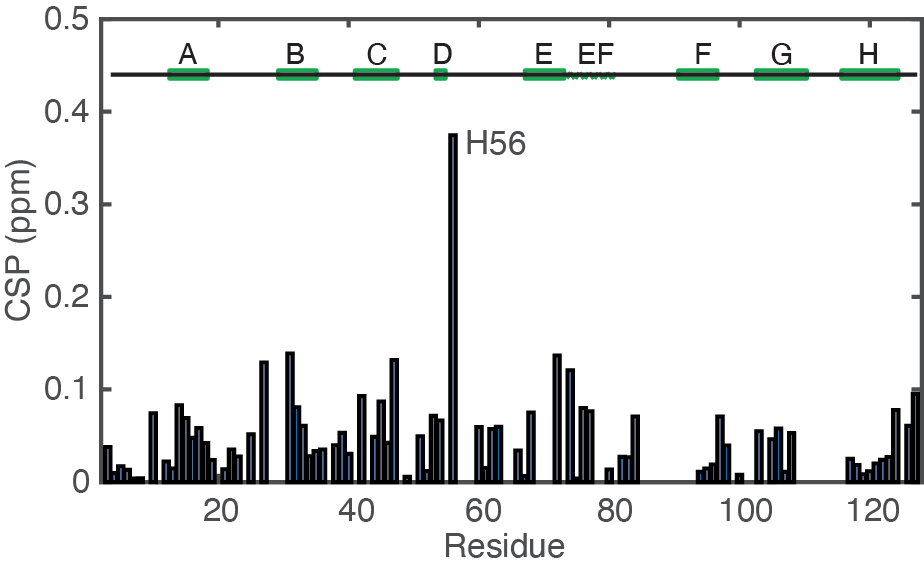


Figure S12. Weighted average chemical shift perturbation (CSP) of F87E at pH 6.7/277 K vs. pH 4.4/277 K. CSP was calculated using $\sqrt{\left( \Delta\delta^{1}H \right)^{2}+\left( \Delta\delta^{15}N/5 \right)^{2}}$.


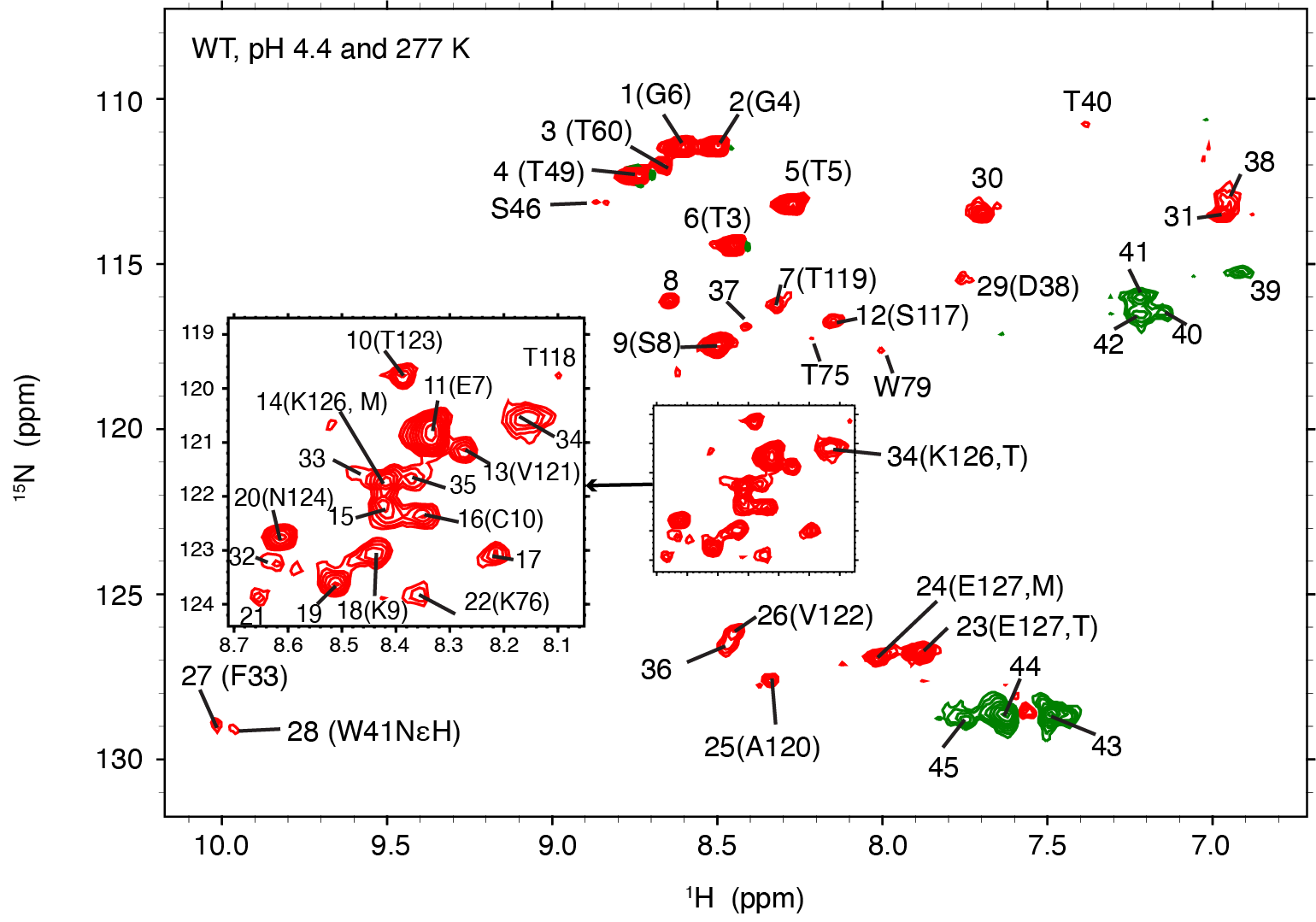


Figure S13. The full ^1^H,^15^N-HSQC spectrum of WT TTR after 180 mins at pH 4.4 and 277 K in the presence of 100 mM KCl. A close-up view of the central region is shown as an inset box on the left. The cross peaks are identified by arbitrary peak numbers corresponding to the data in Figure S14. Positive contours are in red and negative in green.


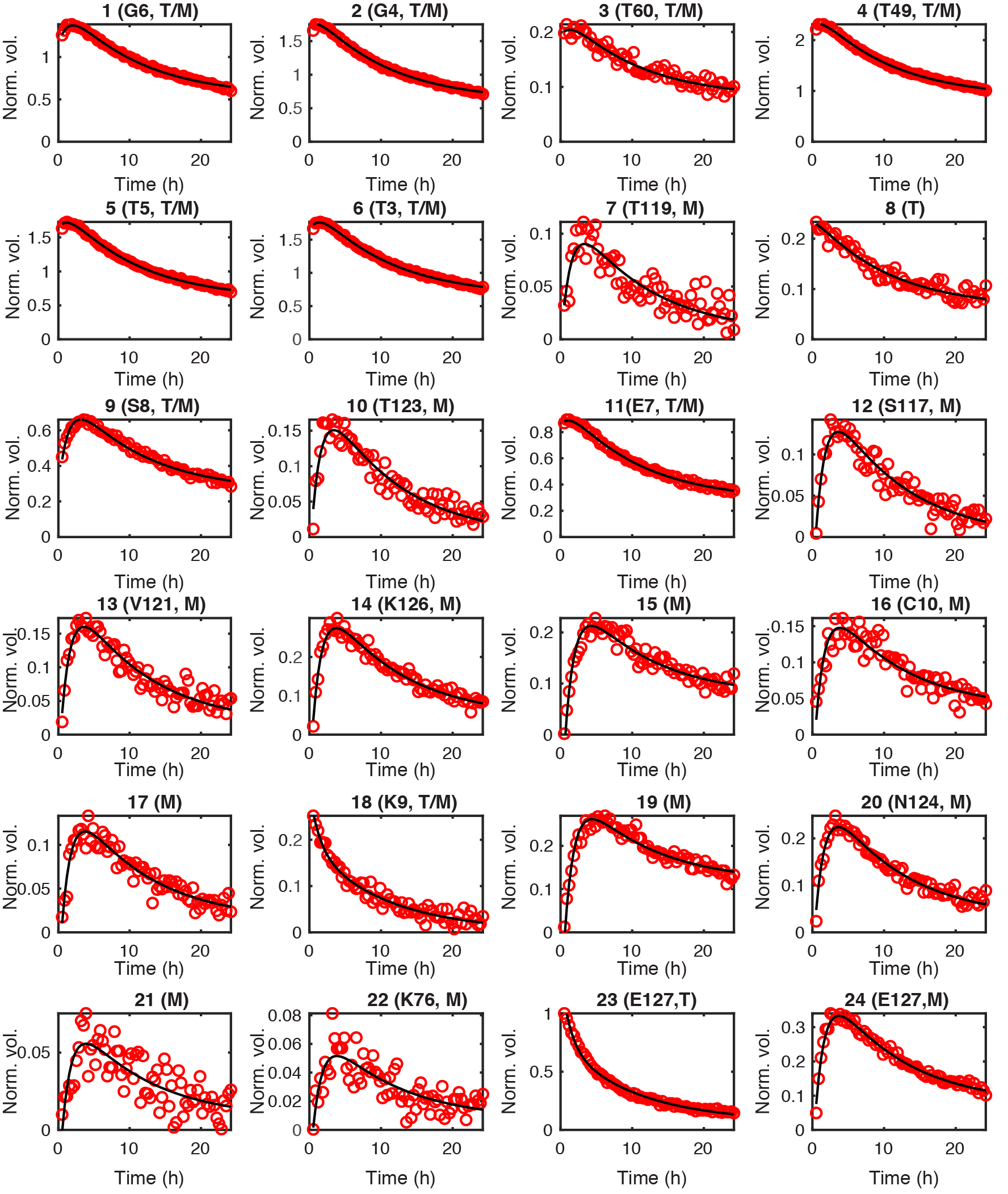


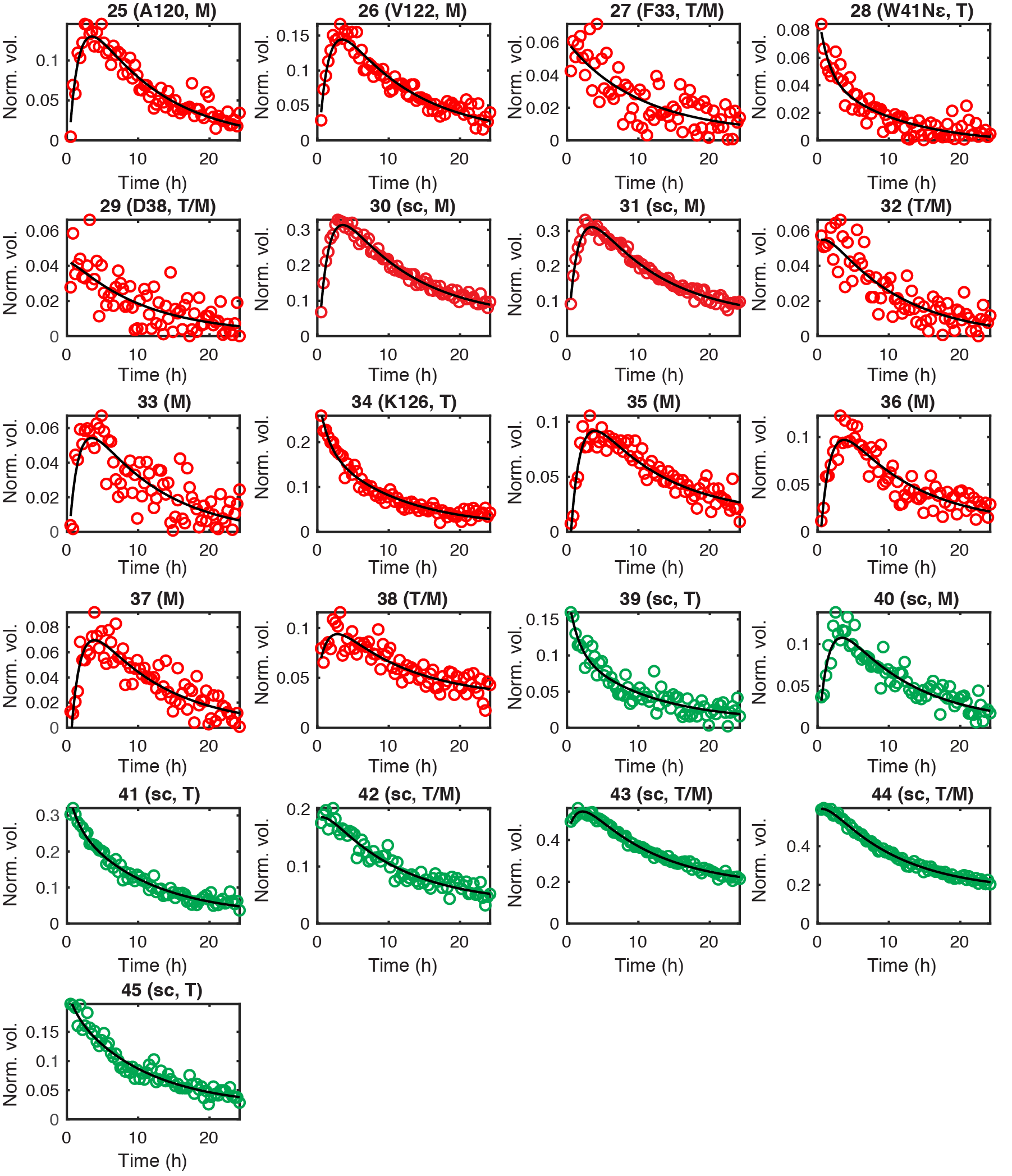


Figure S14. TTR aggregation kinetics probed by backbone (red) and sidechain (sc, green, shown as absolute peak volumes) NH cross peaks of 200 µM WT TTR at pH 4.4 and 277 K. The black lines denote the three-state fit (see Methods). The two fitted global relaxation rates (γ_1_ and γ_2_) are 0.74 ± 0.02 and 0.10 ± 0.01 h^-1^, respectively. The initial volume of the tetrameric E127 cross peak was used for normalization.


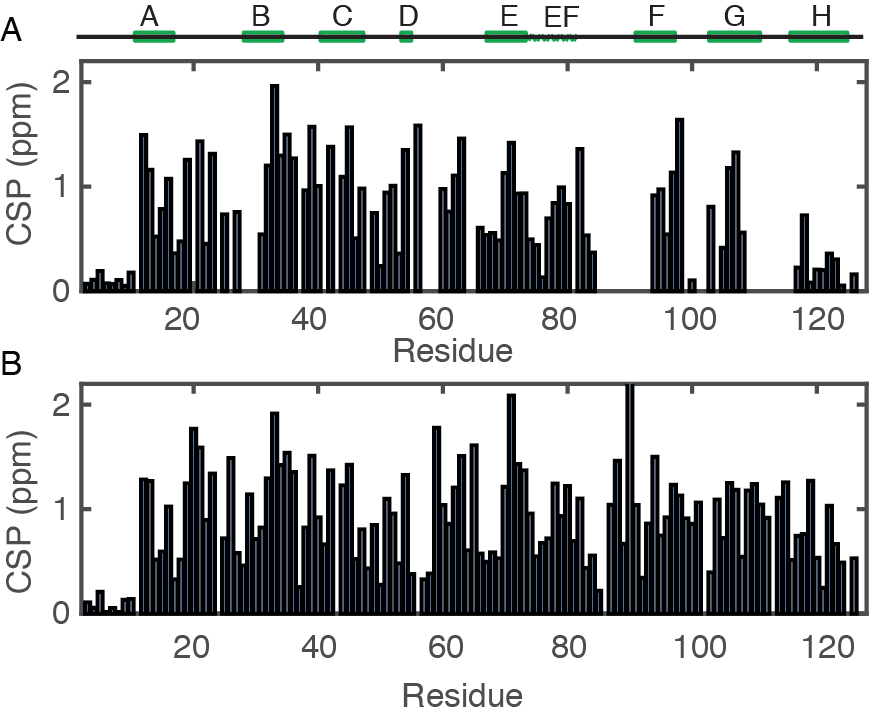


Figure S15. Difference between random coil chemical shifts predicted using the POTENCI server (7) and the experimentally measured chemical shifts of F87E (A, pH 4.4/277 K) and WT TTR (B, pH 6.9/298 K). The absolute value of the chemical shift difference (CSP) is plotted. The chemical shifts of residues in the unfolded N-terminus (T3 to C10) are close to predicted random coil shifts in both monomer and tetramer. The chemical shifts of residues in the C-terminus of the F87E monomer (the H strand) are also close to the predicted random coil chemical shifts and differ substantially from those in the tetramer with a well-folded H strand.


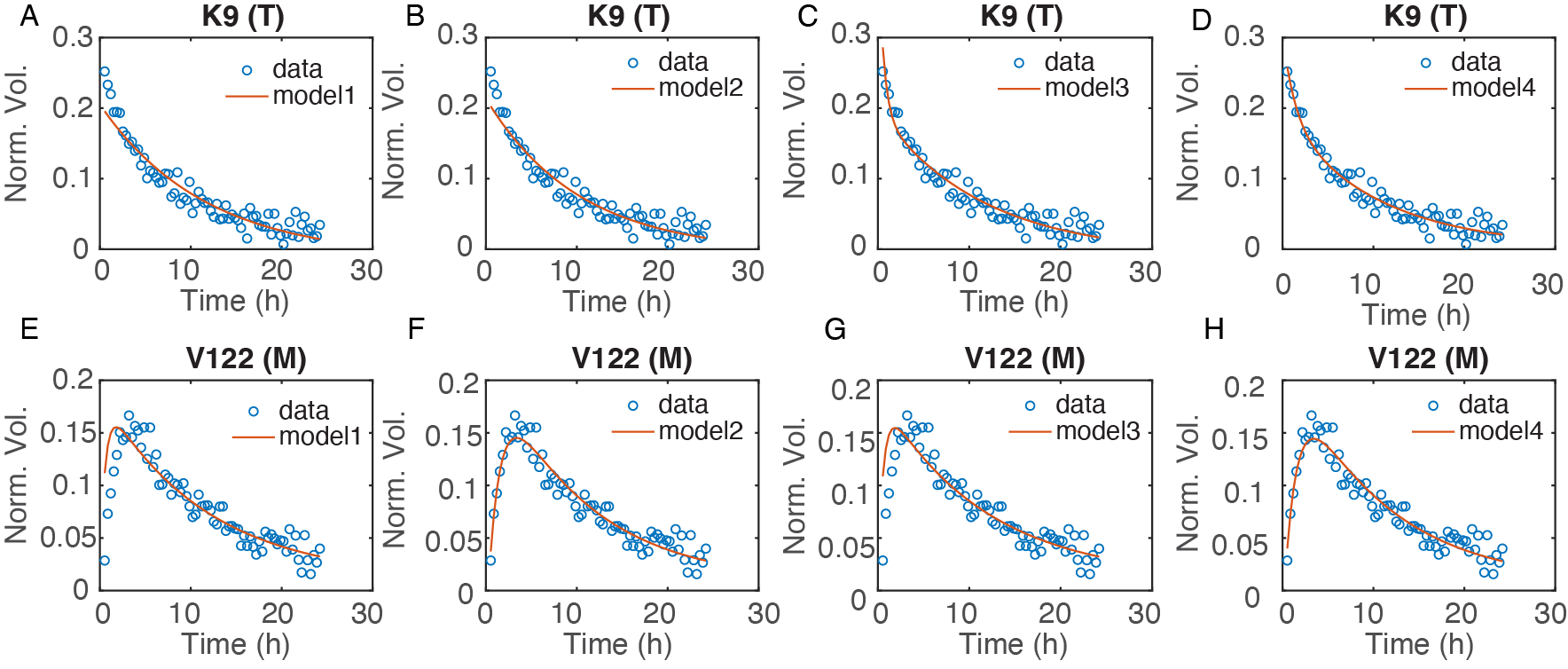


Figure S16. TTR aggregation kinetics probed by backbone amide cross peak volume changes of 200 µM WT at pH 4.4 and 277 K for 1 day (A-D: K9 of the T species, E-H: V122 of the M species). Four models are shown for comparison. Model 1: $T\to M\to A$, Model 2: $T\to M\leftrightharpoons A$, Model 3: $T\leftrightharpoons M\to A$, Model 4: $T\leftrightharpoons M\leftrightharpoons A$. Reversibility in both steps is needed for good fits. Note that models 2 and 4 fit the V122 data but only model 4 provides a good fit to both the V122 and K9 time traces.


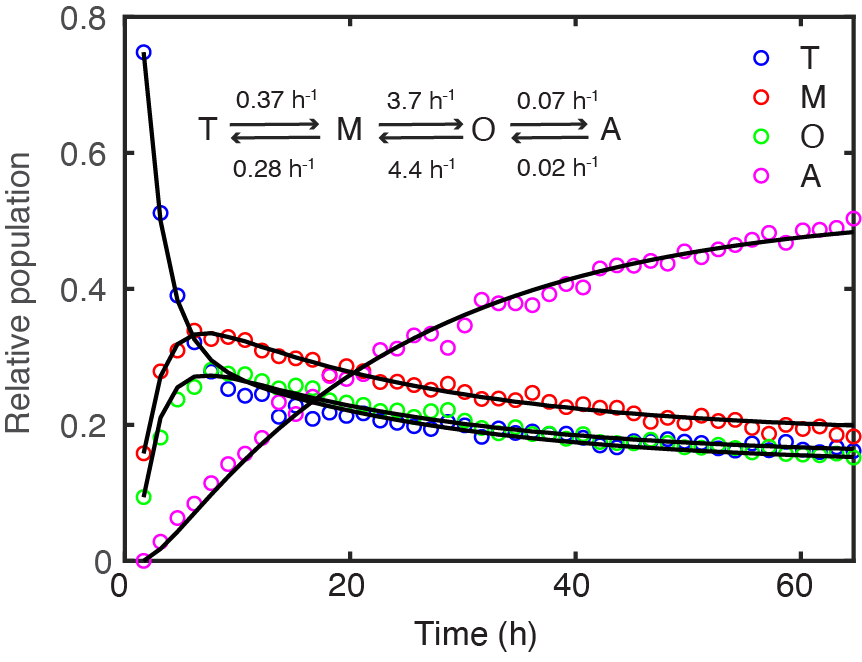


Figure S17. The four-state ($T\leftrightharpoons M\leftrightharpoons O\leftrightharpoons A$) fits for 10 µM S85-TTR^F^ aggregation at 277 K and pH 4.4. The data were taken from Ref (1) and fits are labeled.


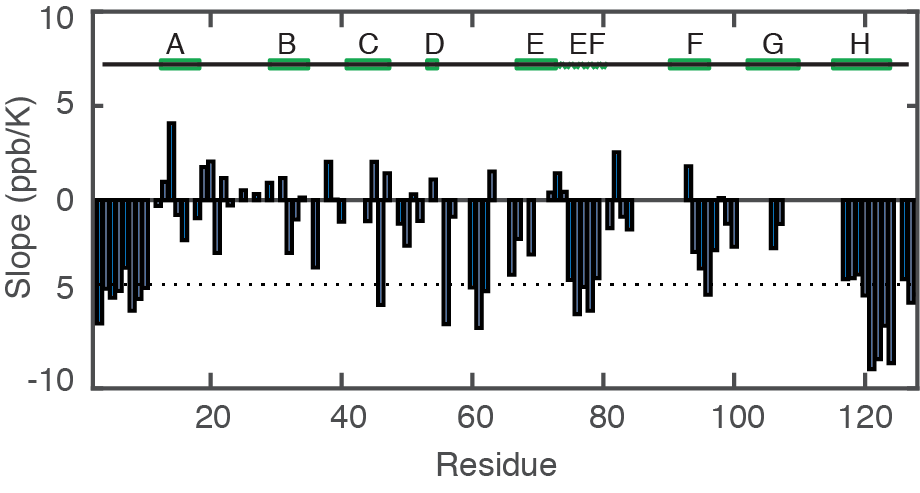


Figure S18. ^1^H temperature coefficients of F87E resonances at pH 6.7. The dashed line denotes 4.5 ppb/K, as the limit to loss of intra-molecular hydrogen-bonds per Ref (8). The most negative slope is from the amide in V121.

**SI References**

1. Sun, X., Dyson, H. J., and Wright, P. E. (2018) Kinetic analysis of the multistep aggregation pathway of human transthyretin. *Proc. Natl Acad. Sci. USA* **115**, E6201-E6208

2. Sun, X., Jaeger, M., Kelly, J. W., Dyson, H. J., and Wright, P. E. (2018) Mispacking of the Phe87 side chain reduces the kinetic stability of human transthyretin. *Biochemistry* **57**, 6919-6922

3. Sun, X., Dyson, H. J., and Wright, P. E. (2017) Fluorotryptophan incorporation modulates the structure and stability of transthyretin in a site-specific manner. *Biochemistry* **56**, 5570-5581

4. Edwards, J. M., Bramham, J. E., Podmore, A., Bishop, S. M., van der Walle, C. F., and Golovanov, A. P. (2019) ^19^F Dark-state exchange saturation transfer NMR reveals reversible formation of protein-specific large clusters in high-concentration protein mixtures. *Anal. Chem.* **91**, 4702-4708

5. Bain, A. D. (2003) Chemical exchange in NMR. *Prog. Nucl. Magn. Res. Spec.* **43**, 63-103

6. Reeves, L. W., and Shaw, K. N. (1970) Nuclear magnetic resonance studies of mutli-site chemical exchange. I. Matrix formulation of the Bloch equations. *Can. J. Chem.* **48**, 3641-3653

7. Nielsen, J. T., and Mulder, F. A. A. (2018) POTENCI: prediction of temperature, neighbor and pH-corrected chemical shifts for intrinsically disordered proteins. *J. Biomol. NMR* **70**, 141-165

8. Baxter, N. J., and Williamson, M. P. (1997) Temperature dependence of ^1^H chemical shifts in proteins. *J. Biomol. NMR* **9**, 359-369
